# Structural engineering of stabilized, expanded epitope nanoparticle vaccines for HPV

**DOI:** 10.3389/fimmu.2025.1535261

**Published:** 2025-01-31

**Authors:** Michaela Helble, Xizhou Zhu, Pratik S. Bhojnagarwala, Kevin Liaw, Yangcheng Gao, Amber Kim, Kelly Bayruns, Madison E. McCanna, Joyce Park, Kylie M. Konrath, Sam Garfinkle, Taylor Brysgel, David B. Weiner, Daniel W. Kulp

**Affiliations:** ^1^ The Vaccine and Immunotherapy Center, The Wistar Institute, Philadelphia, PA, United States; ^2^ Perelman School of Medicine, University of Pennsylvania, Philadelphia, PA, United States

**Keywords:** AI, AlphaFold, MHC-restriction, CTL, HPV, nanoparticle, vaccine, protein design

## Abstract

Oncogenic forms of HPV account for 4.5% of the global cancer burden worldwide. This includes cervical, vaginal, vulvar, penile, and anal cancers, as well as head and neck cancers. As such, there is an urgent need to develop effective therapeutic vaccines to drive the immune system’s cellular response against cancer cells. One of the primary goals of cancer vaccination is to increase the potency and diversity of anti-tumor T-cell responses; one strategy to do so involves the delivery of full-length cancer antigens scaffolded onto DNA-launched nanoparticles to improve T-cell priming. We developed a platform, making use of structural prediction algorithms such as AlphaFold2, to design stabilized, more full-length antigens of relevant HPV proteins and then display them on nanoparticles. We demonstrated that many such designs for both the HPV16 E6 and E7 antigens assembled and drove strong CD8+ T-cell responses in mice. We further tested nanoparticles in a genetically diverse, more translationally relevant CD-1 mouse model and demonstrated that both E6 and E7 nanoparticle designs drove a CD8+ biased T-cell response. These findings serve as a proof-of-concept study for nanoparticle antigen design as well as identify new vaccine candidates for HPV-associated cancers.

## Introduction

Human papillomavirus (HPV) accounts for a staggering 4.5% of the global cancer burden (1). While there are 12 designated carcinogenic HPV strains (2), just two of them, HPV16 and HPV18, are responsible for 72.4% of these cancer cases (1). HPV infection is linked not only to cervical cancer but also to other anogenital cancers such as penile, anal, vulvar, and vaginal, and certain head and neck cancers such as oropharyngeal cancer (1, 3, 4). Human morbidity is propelled by the development of lesions driven by HPV-induced abnormal cell growth (3). While several effective prophylactic vaccines have been developed for HPV (5, 6), there remains an urgent need to develop an effective therapeutic vaccine for those who have established infections.

HPV is a non-enveloped, double-stranded DNA virus (7, 8). The genome encodes for early proteins E1-E7 and late proteins L1-L2. The early proteins control replication of the viral genome while late proteins provide the virion capsid, which then also facilitates entry into other host cells (7–9). While HPV infections can proceed as episomal viral DNA, many patients with established infections have an HPV integration event into the host genome (8, 10, 11). The resultant effect of integration is increased expression of E6 and E7 (12–14), with critical downstream consequences.

E6, in complex with E6AP, binds tumor suppressor p53 and targets it for degradation (15, 16). This leads to unchecked cellular proliferation (7, 13). E7 binds pRb and targets it for degradation (17, 18), which leaves transcription factor E2F free to promote entry into the S phase. Hence, oncogenesis is conferred by E6 and E7 binding to p53 and pRb, respectively. Overall, E6 and E7 levels and their downstream effects as a consequence of p53 and pRb binding lead to highly proliferative cellular growth and resistance to apoptosis, facilitating the malignant transformation of host cells (7, 13). The role of E6 and E7 in driving oncogenesis renders them ideal vaccine targets.

An effective vaccine must be able to generate strong cellular immunity in order to potentiate tumor cell death. Several different approaches have been used to design HPV antigens to try and achieve this, and are summarized nicely in reviews by Yang et al. and Mo et al (19, 20). In general, strategies can be broken into one of four main categories: bacterial/viral vector-based, dendritic cell-based, protein/peptide-based, or nucleic acid-based. Many completed and initiated clinical trials fall into either the protein/peptide-based category or DNA/RNA-based category (20), with other preclinical studies underway. Popular approaches include the use of peptides and synthetic long peptides as combination therapeutics or formulated with adjuvants (20–22). On the nucleic acid side, several clinical trials delivering DNA encoding for E6 and E7 have demonstrated immunity and showed positive initial findings (23–27). For example, a recent phase II trial used an antigen processing-enhanced E7 DNA vaccine in a prime/boost scheme with a recombinant HPV fusion protein and showed an association with viral regression (27). Another phase IIb clinical trial delivering DNA-encoded monomeric E6 and E7 demonstrated significant disease attenuation and viral clearance in the treated groups; this recently met certain phase III clinical endpoints (25, 26).

Building on these important results in the DNA delivery space, it has recently been reported for both infectious disease and cancer applications that DNA-delivered nanoparticles can generate more potent immune responses than some of their corresponding monomeric counterparts (28–30). Here, we exploit this DNA-delivered nanoparticle technology to design improved HPV E6 and E7 immunogens that maximize the number of potential CD8+ epitopes through the delivery of larger, more native-length antigens. This type of larger immunogen display can be difficult; however, with the advent of new AI tools, such as the well-known structural prediction tool AlphaFold2 (31), the ability to design stabilized HPV nanoparticles with more immunogenic epitopes has become simpler. We further test the hypothesis that DNA-delivery and nanoparticle assembly of these stabilized immunogens will drive induction of potent T-cell responses.

To date, the application of AI in cancer has primarily been focused on the development of predictive algorithms, such as improving the accuracy of lesion prediction in pap smears or understanding and altering the tissue distribution of nanoparticles (32–34). To our knowledge, the use of AI toolsets has not yet been applied to aid in the structural design of specific cancer-targeting nanoparticles, nor has *in vivo* immunogenicity been assessed. We describe a generalizable workflow to create potent, stabilized, DNA-launched nanoparticle vaccines and apply this to oncogenic HPV16 proteins E6 and E7 to design more potent vaccine immunogens.

We develop stabilized nanoparticle vaccines of E6 and E7, including versions that lack the ability to bind p53 and pRb, respectively. This is critical for downstream translation. Following *in vitro* characterization, we study the potency of DNA-launched E6/E7 vaccines and observe that multiple nanoparticle designs elicit strong T-cell responses. Furthermore, these nanoparticles are superior at generating CD8^+^ biased T-cell responses over monomeric controls. Importantly, we also assess T-cell responses in outbred mice, which is a better mimic for the HLA diversity of a human population. We demonstrate that a combination E6/E7 nanoparticle vaccine generates responses, even in this genetically diverse mouse population. These results represent an important step in designing next-generation nanoparticle cancer vaccines.

## Results

### Stabilized nanoparticle design workflow

**Figure 1:**
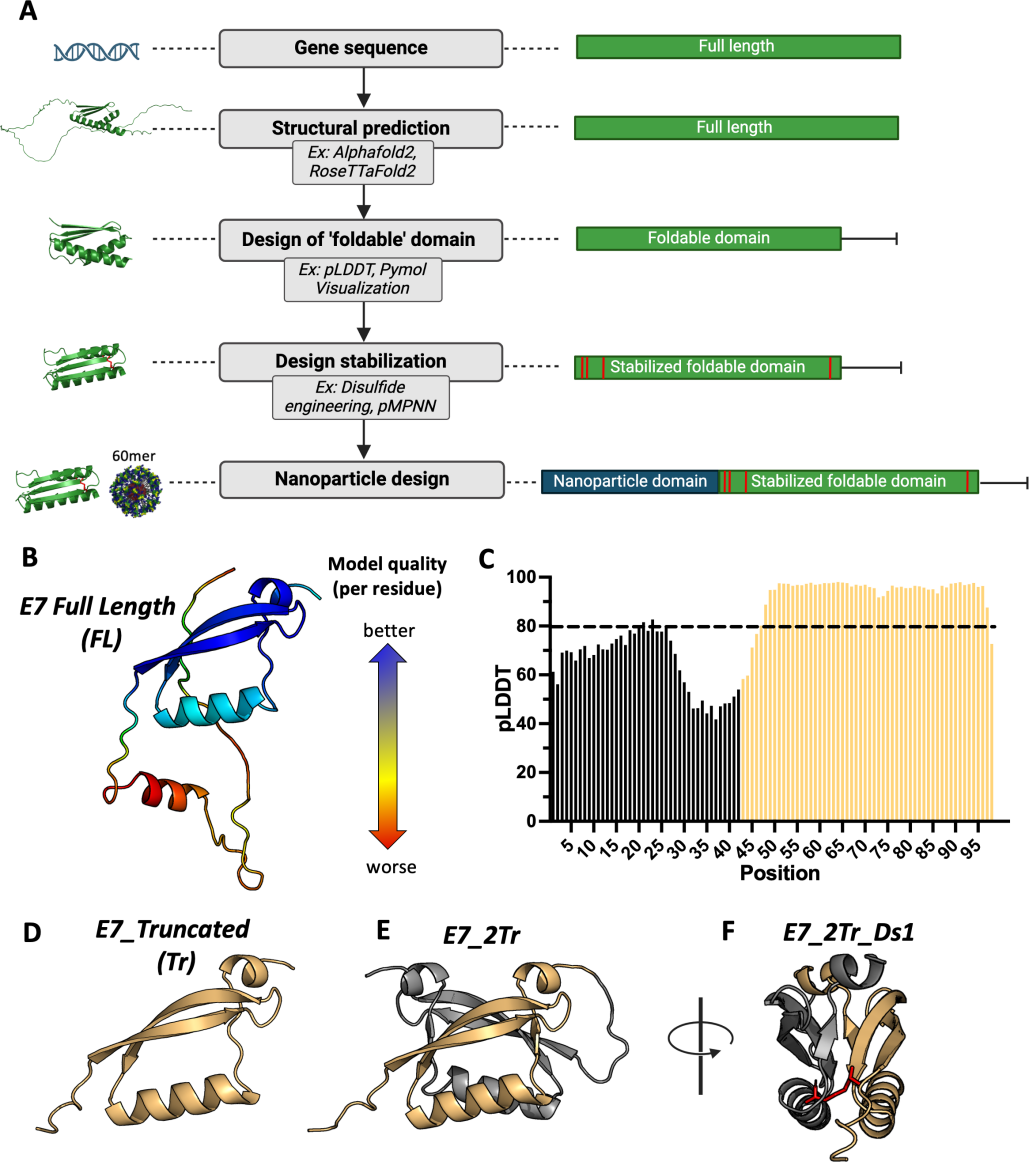
Stabilized nanoparticle design workflow. **(A)** The desired protein sequence is run through structural prediction algorithms such as AlphaFold2 and RoseTTafold2. Confidence metrics associated with the structural prediction help determine ordered regions from disordered regions, along with visual inspection of the predicted structure. This results in the design of a minimized ‘foldable’ domain. Additional design stabilization can be engineered in; here, we use disulfide scanning to introduce pairs of disulfide bonds to stabilize local folds. Finally, the resultant candidate designs are scaffolded onto a self-assembling 60-mer nanoparticle scaffold; **(B)** Example of structural prediction. The top-ranked model of WT E7 from RoseTTaFold2, colored by per-residue RMS-error. Red residues are lower confidence while blue residues are higher confidence; **(C)** Example of a confidence metric graph. pLDDT scores from the top ranked Alphafold2 prediction score of WT E7 is depicted. Scores above 80 are considered high confidence and guide selection of structurally intact domains. Residues that comprise the selected foldable domain are shown in yellow; **(D)** Example of a selected foldable domain. Using confidence metric scores and visual inspection, residues that comprise a purported foldable domain were selected. The sequence was then re-predicted to confirm purported structural integrity, and the top ranked AlphaFold2 prediction is shown; **(E)** Example of an iterative foldable domain design. WT E7 can form a dimer. This guided creation of a covalent dimer of the truncated domain design in **(D)**. The top ranked Alphafold2 prediction of the designed dimeric sequence is shown; **(F)** Example of addition design stabilization. A disulfide scanning algorithm was used to select pairs of residues that could be mutated to cysteine to form a new disulfide bridge. Here, the scanner was applied to find disulfide pairs that bridge the dimeric interface to create additional stabilization of the fold. The engineered disulfide bridge is depicted in stick representation and colored red.

Antigens with maximal epitopes may be able to generate broader T-cell responses. An ideal vaccine candidate would therefore be designed to display these more full-length antigens on a DNA-launched, 60-mer nanoparticle scaffold. This type of scaffold provides stronger T-cell priming and more potent effector immune responses (28–30). With this in mind, we sought to develop a cancer antigen design workflow utilizing the power of AI and computational design tools (**Figure 1A**). The developed workflow consists of 5 steps: determination of gene sequence, structural prediction, design of a ‘foldable’ domain, additional design stabilization, and formulation as a nanoparticle.

Once the gene sequence for the desired antigen is determined, a predicted structure for the full-length antigen is generated through prediction tools such as RoseTTafold2, AlphaFold2, or the newer AlphaFold3 (31, 35, 36). In some cases, the full-length structure may not have well-folded domains, which could interfere with display. Confidence metrics from structural prediction algorithms, such as Alphafold’s pLDDT score, have been shown to correlate with well-folded regions (37, 38). Loading the predicted structures into structure visualization tools like PyMOL, coupled with confidence metric scores, allows for the selection of residues in a truncated, purported ‘foldable’ domain in the event that the full-length antigen cannot be displayed. Additional stabilization can be engineered into these constructs. One way to do so is through the use of disulfide engineering, in which pairs of residues are mutated to cysteines to create a covalent disulfide bridge that can stabilize local folds. ProteinMPNN (39) could be used at this stage to change residues with the hope of achieving display, though there is the tradeoff that less native-like sequences will be inferior at achieving robust anti-cancer T-cell responses. Finally, candidate designs are scaffolded onto a stabilized, lumazine-synthase domain that creates self-assembling 60-mer nanoparticles *in vivo* following gene delivery, as previously described (28). Any construct that is a 60-mer nanoparticle will contain a ‘nano’ designation throughout.

To test this design approach, we focused on the development of HPV16 E7 as a model antigen for a proof-of-concept study, as it is a well-described, important target for HPV vaccine efforts (7). Example outputs from structural prediction algorithms and examples of per residue confidence metrics are shown for full-length E7 (E7_FL) (**Figures 1B, C**; **Supplementary Figures S1A-D** **).** Comparison of the structure with per-residue pLDDT scores showed high confidence for the C-terminal domain folds, which we used as the basis for creating a truncated, foldable domain. We used a cutoff of pLDDT >= 80 as this falls into the confident-very confident prediction score range to down-select residues (31, 40). Though residues 43-46 have pLDDT scores lower than this cutoff, we preserved them in our truncated structure to create a more native-like linker when scaffolded onto the nanoparticle core. The resultant structure that forms the basis of the E7 truncated foldable domain (E7_Tr) is shown in **Figure 1D**. An NMR partial structure of E7 for a different strain (HPV45) showed E7 in a dimeric form (PDB: 2F8B). Single subunits of the resolved dimer have a highly similar structure to our designed E7_Tr (**Supplementary Figure S1E**). We therefore reasoned that E7_Tr was likely to be stable as a dimer but with the potential to be more immunogenic due to a higher number of domains, so we formulated a dimeric version (E7_2Tr) with a flexible GS linker between individual subunits (**Figure 1E**). In order to further stabilize the dimeric form, we chose to use a disulfide scanner algorithm (see Methods) to select pairs of residues amenable to mutation to cysteine in order to staple the dimeric interface together (**Figure 1F** **).** While disulfide engineering is not the only tool that can be used to achieve protein stabilization (41, 42) we chose to focus on this method as stabilization can be achieved with minimal mutations (43–45). We also used this method to create pairs of disulfides that might stabilize local folds in the full-length structure (E7_FL) to promote expression on a nanoparticle, as well as to E7_Tr in the event domain minimization was not sufficient to achieve display. We generated three disulfide modified full-length versions (E7_FL_Ds1, E7_FL_Ds2, E7_FL_Ds3), a truncated version (E7_Tr_Ds1), and a truncated dimer version (E7_2Tr_Ds1). Sequences for designs can be found in **Supplementary Figure S2**. All E7 designs were then formulated onto the self-assembling 60-mer scaffold to create nanoparticles.

### *In vitro* characterization of E7 nanoparticle designs

**Figure 2:**
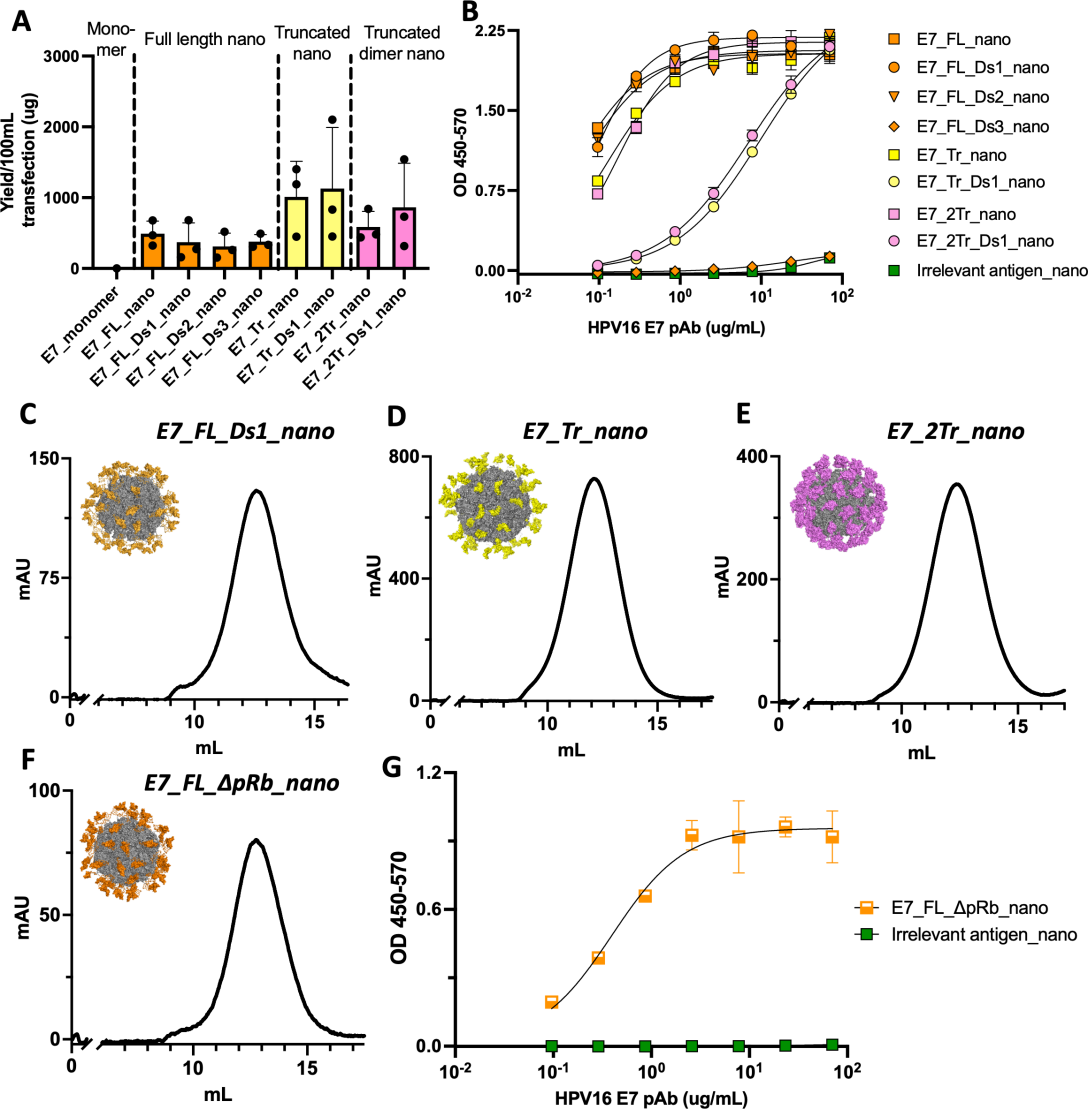
*In vitro* characterization of E7 designs. **(A)** Purified transfection yield from Expi293F cells for designed E7 nanoparticles. Nanoparticles are grouped by their base design: full length, truncated, or truncated dimer. Transfection volume was 100mL and constructs were purified by lectin affinity chromatography and size exclusion chromatography before yield was determined; **(B)** Binding of designed E7 nanoparticles to an HPV16 E7 polyclonal antibody. Plates were coated with 15μg/mL of each nanoparticle followed by incubation with the serially diluted polyclonal antibody at the concentrations indicated; Size exclusion chromatography traces (215nm) of E7 nanoparticles with models in the inset for **(C)** E7_FL_Ds1_nano; **(D)** E7_Tr_nano; **(E)** E7_Tr_Dim_nano; **(F)** E7_FL_ΔpRb_nano; **(G)** Binding of E7_FL_ΔpRb_nano to an HPV16 E7 polyclonal antibody. Plates were coated with 15μg/mL of each nanoparticle followed by incubation with the serially diluted polyclonal antibody at the concentrations indicated.

The biophysical characterization of E7 nanoparticle designs was first assessed *in vitro*. We saw that all nanoparticle designs were easily able to form *in vitro* in contrast to E7_monomer (0μg yield), showcasing the power of the nanoparticle platform to aid in design expression and stabilization (**Figure 2A**). The full-length nanoparticle designs had the lowest average purified yields, ranging from 311.5-490.1μg from a 100mL transfection. This was expected as all E7_FL nanos contained a large unstructured portion according to the structural prediction algorithms, which would likely reduce their ability to form stably. In this case, the introduction of disulfide bonds through E7_FL_Ds1, Ds2, or Ds3 nanos did not have a large effect on the yield. In contrast, designs based on E7_Tr or E7_2Tr both had higher average yields than designs based on E7_FL, and disulfides further increased the yield, showcasing the importance of introducing structure-guided truncations. The average yield of E7_Tr_nano was 1012.3μg and 1128.3μg for E7_Tr_Ds1_nano; E7_2Tr_nano had an average yield of 586.5μg, and E7_2Tr_Ds1_nano had an average yield of 862μg.

To assess the structural integrity of the E7 antigens displayed on nanoparticles, we performed ELISAs to determine binding to a polyclonal anti-E7 antibody. Most designs bound well, showing proper antigen formation (**Figure 2B**). Though other designs such as E7_Tr_Ds1_nano and E7_2Tr_Ds1_nano formed well *in vitro*, they had reduced ELISA binding suggesting that some of the disulfides introduced might cause partial antigen misfolding.

We weighed *in vitro* yield as well as antigen structural integrity to decide which nanoparticles to use in a first-pass immunogenicity experiment. We first chose one nanoparticle from each base construct category; size exclusion chromatography (SEC) traces further confirm their formation as nanoparticles and models of each 60-mer are shown in the insets (**Figures 2C–E**). E7_FL_Ds1_nano was chosen in the full-length category as it had similar yields to E7_FL_nano but slightly better ELISA binding. E7_Tr_nano and E7_2Tr_nano were both selected over their disulfide-modified counterparts due to the better structural integrity of these antigens. Ultimately E7_2Tr_nano was chosen over E7_Tr_nano as they both contain the same truncated domains, but E7_2Tr_nano has twice as many of these domains, meaning that it might generate an even more potent immune response.

To assess whether E7_FL_Ds1_nano and E7_2Tr_nano could elicit T-cell immunity, we immunized mice and analyzed splenocytes post-vaccination. We observed that both nanoparticle groups mounted a significant T-cell response as determined by ELISpot (**Supplementary Figures S3A, B**).

### Formulation of non-oncogenic E7 nanoparticles

Encouraged by this immunogenicity experiment, we decided to iterate on our initial designs to create non-oncogenic versions of these nanoparticles. E7 has oncogenic properties, conferred by its ability to bind pRb and initiate pro-tumorigenic downstream effects (7). A safer vaccine candidate would be pRb binding null (and indeed, this mirrors other vaccines found in clinical trials) (46). The location of the truncation found in E7_2Tr_nano is such that this design naturally lacks pRb binding, and so no changes had to be made for this design. However, we decided to introduce pRb binding knockout mutations (hereafter ΔpRb) (46) into E7_FL_nano over E7_FL_Ds1 to maximize native-like epitopes. This became E7_FL_ΔpRb_nano (**Supplementary Figure S2**). Though several options of mutations to knock out pRb binding exist, we chose pRb-knockout mutations derived from VGX-3100, as products incorporating these mutations have advanced to clinical trials (25, 26, 46, 47).

Alphafold2 predictions showed no major changes to the structure introduced by the ΔpRb mutations, confirming this was a viable new design (**Supplementary Figure S4A**). We confirmed the *in vitro* formation of the design as well as the assembly of E7_FL_ΔpRb_nano into a 60-mer via SEC (**Figure 2F**, **Supplementary Figure S4B**). A model of the resultant nanoparticle is shown in the inset. The structural integrity of E7_FL_ΔpRb_nano was also assessed by ELISA binding to the polyclonal anti-E7 antibody; antigen integrity was preserved (**Figure 2G**). This provided two final E7 nanoparticle candidates of interest for more extensive *in vivo* immunogenicity experiments: E7_FL_ΔpRb_nano and E7_2Tr_nano; both of which lack binding to pRb for safety considerations. Their formation as nanoparticles was further confirmed by negative stain electron microscopy (nsEM) and is consistent with the assembly of other 60-mer lumazine synthase nanoparticles displaying alternative antigens (28, 29)(**Supplementary Figures S5A, B**).

### *In vitro* characterization of E6 nanoparticle designs

As the nanoparticle design workflow should be easily adaptable to other antigens, we tested this idea by also engineering E6-targeting nanoparticles. Both E6 and E7 have been the targets of many vaccination efforts since they both are oncogenic and are constitutively expressed (7, 19, 23, 25). In the case of HPV16 E6, a resolved crystal structure (PDB: 6SJA) is available, though it contains mutations relative to WT that enhance solubility and reduce aggregation (hereafter referred to as ‘Sol’ mutations). We used structural prediction algorithms to confirm that the WT sequence of full-length HPV16 E6 (E6_FL) was predicted to fold in a similar manner to the crystal structure (**Supplementary Figure S6A**); they are essentially identical and fold into an N-terminal and C-terminal domain separated by an alpha helix.

Ordinarily, the next step in the nanoparticle design workflow would be to select a truncated domain solely composed of regions predicted to be well folded. However, E6 has very high per residue pLDDT scores across the full protein (**Supplementary Figure S6B**), reflecting its well-folded nature. We instead decided to create a smaller, truncated domain from structural visualization and based on residues 7-87 in the event that the entire full-length protein was too large to display properly as a nanoparticle. This design became E6_Tr. We further reasoned that the Sol mutations might be necessary for stability and formation as they were integral to crystal structure resolution, so we engineered versions of E6_FL and E6_Tr with Sol muts (E6_FL_Sol and E6_Tr_Sol). Both full-length and truncated E6 designs with additional disulfides engineered were also created, resulting in three disulfide-engineered full-length variants (E6_FL_Ds1, E6_FL_Ds2, E6_FL_Sol_Ds1) and two disulfide-engineered truncated variants (E6_Tr_Ds1, E6_Tr_Ds2). Sequences for all designs can be found in **Supplementary Figure S7**. Finally, all designs were formulated onto our self-assembling 60-mer scaffold to create nanoparticles.

**Figure 3:**
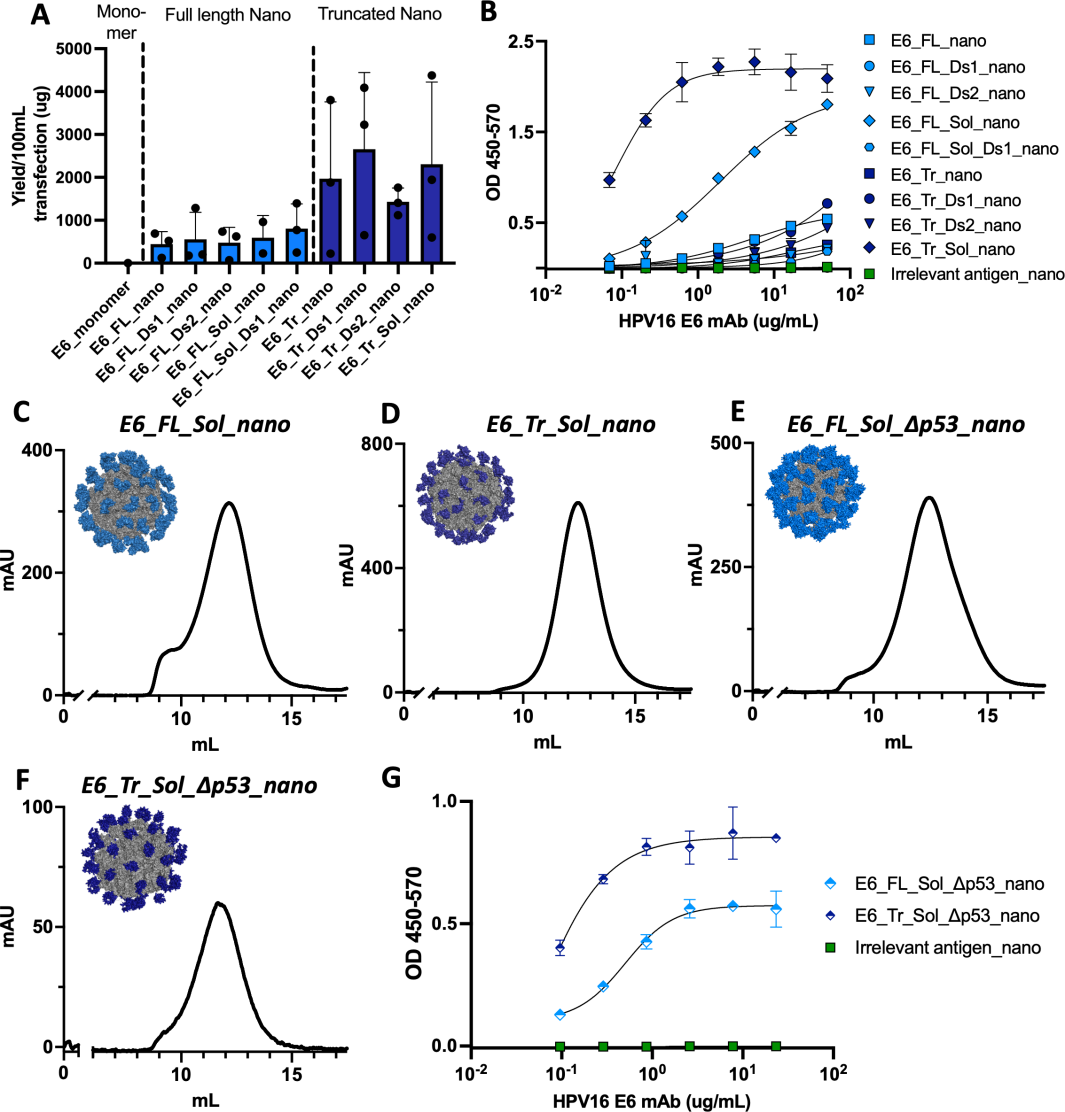
*In vitro* characterization of E6 designs. **(A)** Purified transfection yield from Expi293F cells for designed E6 nanoparticles. Nanoparticles are grouped by their base design: full length or truncated. Transfection volume was 100mL and constructs were purified by lectin affinity chromatography and size exclusion chromatography before yield was determined; **(B)** Binding of designed E6 nanoparticles to an HPV16 E6 antibody. Plates were coated with 15μg/mL of each nanoparticle followed by incubation with the serially diluted antibody at the concentrations indicated; Size exclusion chromatography traces (215nm) of E6 nanoparticles with models in the inset for **(C)** E6_FL_Sol_nano; **(D)** E6_Tr_Sol_nano; **(E)** E6_FL_Sol_Δp53_nano; **(F)** E6_Tr_Sol_Δp53_nano; **(G)** Binding of Δp53 E6 nanoparticles to an HPV16 E6 antibody. Plates were coated with 15μg/mL of each nanoparticle followed by incubation with the serial diluted antibody at the concentrations indicated.

This suite of designs was assessed for *in vitro* expression. All formed well *in vitro* in contrast to the WT E6 monomer (E6_monomer, 0μg yield), demonstrating that the nanoparticle scaffold aids in design expression and stabilization (**Figure 3A**). Interestingly, the Sol and disulfide modifications made little difference in overall yield; yield instead changed according to full-length constructs (lower yield) or truncated constructs (higher yield). Designs based on the full-length E6 sequence had average purified yields of 443.2-805.5μg, while designs based on the truncated domain had average purified yields of 1429.6μg-2656.6μg.

ELISA binding to an HPV16 anti-E6 antibody was used as a proxy to assess the structural integrity of each nanoparticle antigen (**Figure 3B**). In this case, Sol mutations were key to maintaining structural integrity. Only E6_FL_Sol_nano and E6_Tr_Sol_nano displayed meaningful binding to the anti-E6 antibody. SEC traces of these two constructs further demonstrate their formation as nanoparticles and models of each 60-mer are shown in the insets (**Figures 3C, D**).

To assess whether E6_FL_Sol_nano and E6_Tr_Sol_nano were not just structurally intact, but also capable of mounting a relevant immune response, we immunized mice and assessed responses. We observed immune responses generated by both nanoparticles, with higher responses in mice immunized with E6_Tr_Sol_nano (**Supplementary Figures S3A**, **S4C** **).**

### Formulation of non-oncogenic E6 nanoparticles

Akin to E7, the oncogenic properties of E6 are driven by its ability to bind another protein, in this case, tumor suppressor p53. In order to create versions without these potential oncogenic properties, we introduced mutations and deletions in the p53 binding region. This also corresponds with established p53 knockout mutations incorporated in products that have advanced to clinical trials (25, 26, 46, 47). The set of changes to knock out p53 binding will be referred to as Δp53. We accordingly engineered two new nanoparticles without functional oncogenic sequences, E6_FL_Sol_Δp53_nano and E6_Tr_Sol_Δp53_nano (**Supplementary Figure S7**). Alphafold2 models of the new designs showed that designs incorporating Δp53 mutations are predicted to maintain similar folds to the crystal structure (**Supplementary Figures S4C, D**).

*In vitro* formation of the designs, as well as the proper assembly of both nanoparticles, were confirmed (**Figures 3E, F**, **Supplementary Figure S4E**). Models of the new nanoparticles demonstrate similarities between E6_FL_Sol_nano and E6_FL_Sol_Δp53_nano, as well as between E6_Tr_Sol_nano and E6_Tr_Sol_Δp53_nano, as predicted. Finally, the structural integrity of E6_FL_Sol_Δp53 and E6_Tr_Sol_Δp53_nano was assessed by ELISA binding to an anti-E6 monoclonal antibody. Binding was preserved with identical binding patterns to the E6 designs with intact p53 binding; the truncated antigen displayed superior binding to the full length (**Figure 3G**). These results provided E6_FL_Sol_Δp53_nano and E6_Tr_Sol_Δp53_nano as designs to move forward for more extensive *in vivo* immunogenicity experiments, and their formation as nanoparticles was further confirmed by nsEM (**Supplementary Figures S5C, D**).

### T-cell responses induced by designed E7 nanoparticles

**Figure 4:**
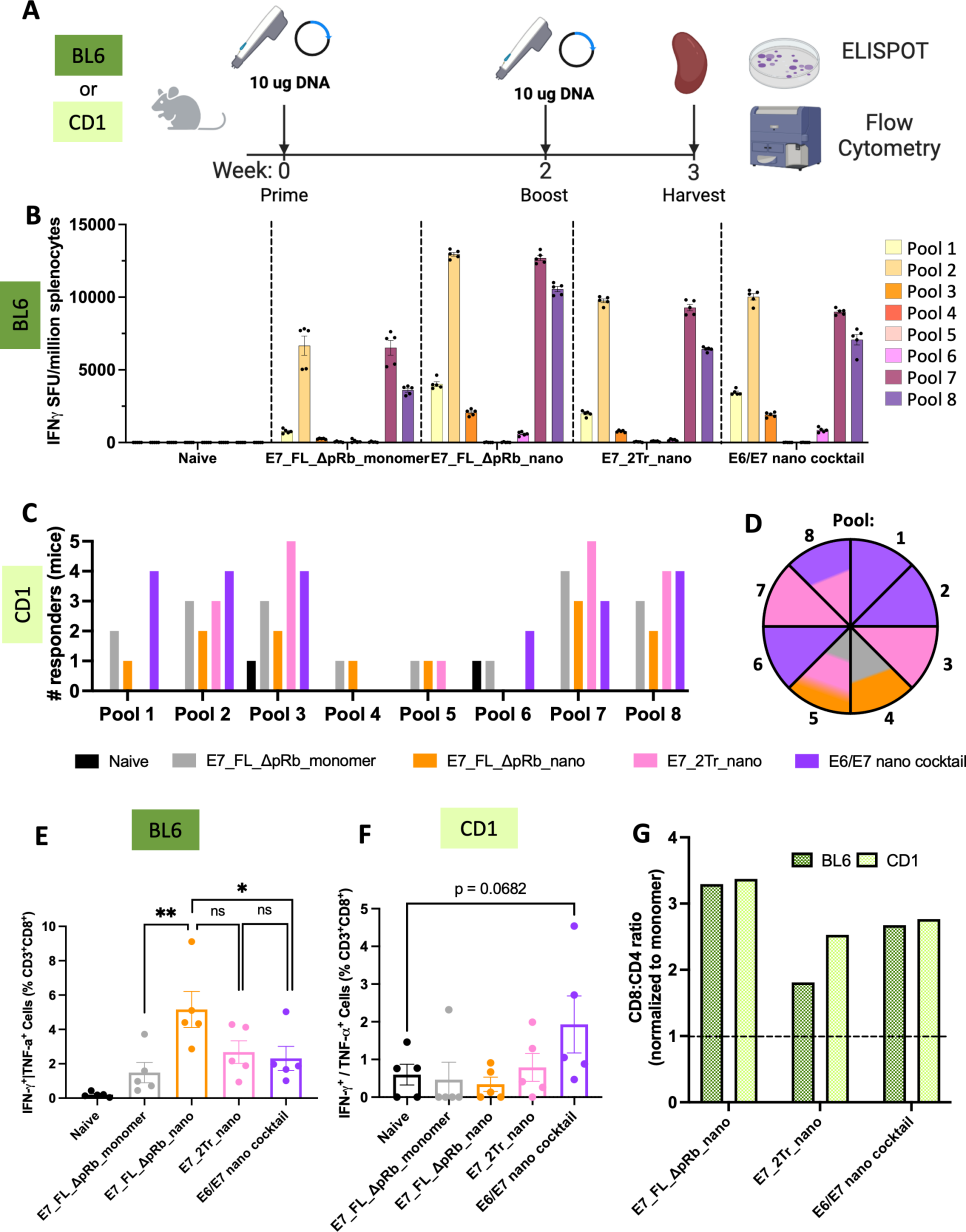
T-cell responses of designed E7 nanoparticles. **(A)** Immunizations overview. Either C57/BL6 or CD-1 mice were immunized with 10μg of DNA (IM-EP) at week 0. Animals were boosted with an equivalent dose at week 2, and their spleens were harvested at week 3. Splenocytes were isolated for downstream use in ELISpot and flow cytometry experiments; **(B)** ELISpot responses in C57/BL6 mice. Splenocytes from immunized mice were stimulated with overlapping peptide pools that span WT E7. IFNγ spot-forming units per million splenocytes are quantified. (n=5 mice/group). Mean ± SEM; **(C)** Positive T-cell responders in CD-1 mice. ELISpot responses were quantified as above, with additional normalization to the average positive SFUs in the naïve group. Any mouse retaining strong responses after normalization to naïve was counted as a responder. The number of responders per pool per immunization group was determined. (n=5 mice/group). Black = naïve, gray = E7_FL_ΔpRb_monomer, orange = E7_FL_ΔpRb_nano, pink = E7_2Tr_nano, purple = E6/E7 nano cocktail; **(D)** Top immunogenic constructs per pool in CD-1 mice. The pool number is indicated per wedge, and colored according to the immunization group that had the highest number of mice responding for that pool. In cases where multiple constructs had the same number of responders, the wedge color is split accordingly. The color scheme is identical to **(C)**; **(E)** IFNγ^+^ or TNFα^+^ CD8^+^ T-cell responses in C57/BL6 mice. Splenocytes were stimulated with E7 peptides and protein transport inhibitors, and CD8^+^ T-cell responses were quantified by intracellular cytokine staining and flow cytometry. Mean ± SEM shown. One-sided T-tests were conducted for group comparisons of interest. All groups are significant compared to naïve; labels were omitted for ease of viewing. ns: not significant, *p<0.05, **p<0.005; **(F)** IFNγ^+^ or TNFα^+^ CD8^+^ T-cell responses in CD-1 mice. Splenocytes were analyzed as above. Mean ± SEM shown. One sided T-tests were conducted; **(G)** Nanoparticle T-cell bias. CD8^+^:CD4^+^ T-cell ratios were determined as the average of (%) IFNγ^+^ or TNFα^+^ CD8^+^ T-cells/(%)IFNγ^+^ or TNFα^+^ CD4^+^ T-cells per group. These were normalized to the average CD8^+^:CD4^+^ ratio for the E7 monomer to determine the fold CD8^+^:CD4^+^ stimulation bias for nanoparticle groups over monomer. Anything above the dotted line is superior to monomer.

Mice were immunized with 10μg of DNA in a prime/boost immunization scheme (**Figure 4A**). Spleens were harvested one-week post boost and used to determine the immunogenicity of the DNA-launched nanoparticle designs via interferon-γ ELISpot and intracellular cytokine staining by flow cytometry. E7_FL_ΔpRb_nano and E7_2Tr_nano were compared to a monomeric construct, E7_FL_ΔpRb_monomer, in order to ascertain if nanoparticle responses were superior to monomer. Further, because of the suggestion that E6 and E7 responses can synergize, a group consisting of E6_Tr_Sol_Δp53_nano and E7_2Tr_nano was also assessed (hereafter ‘E6/E7 nano cocktail’).

Immunogenicity was first analyzed for inbred C57/BL6 mice (**Figure 4B**). All monomeric and nanoparticle groups displayed E7-specific T-cell responses. As expected, all nanoparticle groups were also able to elicit stronger E7-directed immunogenicity than the monomer. The monomer had an average of 6,666 spots for the highest-responding E7 peptide pool 2, compared to average spots of 9,752, 10,030, or 12,957 for E7_2Tr_nano, E6/E7 nano cocktail, and E7_FL_ΔpRb_nano respectively. Across all groups, the strongest responses were directed at pools 2, 7, and 8, reflecting the genetically identical nature of inbred mice. These pools contain the known MHC D^b^-restricted immunodominant peptide, RAHYNIVTF, for BL6 mice (48). Interestingly, though E7_FL_ΔpRb_nano had the lowest *in vitro* expression, we observed the highest magnitude of E7-directed immunogenicity. It may be that the partially unfolded nature of the full-length protein is in fact advantageous in the case of intracellular processing onto MHCI, though this may depend on the exact mechanisms contributing to DNA-primed cellular immunity.

We next assessed immunogenicity in a genetically heterogeneous population as this would be more reflective of the variable nature of responses in a human population. We immunized outbred CD-1 mice, a mouse model with more diverse MHC haplotypes, according to the same immunization scheme (**Figure 4A**). As expected, responses in outbred mice were much more variable due to this genetic diversity (**Supplementary Figure S8A**). In order to better compare overall responses and determine if our designed nanoparticles were eliciting responses to multiple different E7 epitopes, we characterized each CD-1 mouse as a responder or non-responder on a per-pool basis. Responders were normalized to naïve background, which appeared higher in CD-1 mice. Therefore for stringency, mice were required to have >300 spots after normalization to be considered positive responders. Both the monomer and nanoparticle groups were able to achieve responses to a variety of epitopes, as shown by the diversity of pools with positive responders (**Figure 4C**). The group that elicited the highest number of responders on a per-pool basis was determined (**Figure 4D**); each pool is colored according to the group with the most responders. The E6/E7 nano cocktail group had the highest responders in pools 1, 2, and 6 and was tied for pool 8. This group was able to achieve the highest number of responders across diverse pools, suggesting the synergy of immune responses in the cocktail group. The E7_2Tr_nano group was a close second with the highest responses in pools 3 and 7 and tied for pools 5 and 8. Together, these demonstrate the ability of the designed stabilized nanoparticles to elicit immune responses in a genetically diverse background.

To more directly assess whether the nanoparticle groups were priming a cytotoxic T-lymphocyte (CTL) response, we determined the degree of IFNγ^+^ or TNFα^+^ CD8+ T-cell responses (**Figure 4E**). In inbred BL6 mice, a strong CD8+ T-cell response was observed, especially in the E7_FL_ΔpRb_nano group, where an average of 5.16% of the CD8^+^ cells were IFNγ^+^ or TNFα^+^. This corresponds with the ELISpot response data. In outbred CD-1 mice, the E6/E7 nano cocktail has the highest levels of CD8^+^ cells (**Figure 4F**) with an average of 1.93% IFNγ^+^ or TNFα^+^ CD8^+^ T-cells, reflecting the high responder numbers observed in the ELISpot data.

We were interested in determining to what degree the observed nanoparticles bias a CTL over T-cell helper response, ie to what degree responses were CD8^+^ biased over CD4^+^ biased. We observed strongly enhanced CD8^+^:CD4^+^ responses in both inbred and outbred mice for the nanoparticle groups (**Supplementary Figure S8C**) and enhanced abilities to prime CD8^+^ cells over monomer (**Figure 4G**). Average CD8^+^ bias ranges from 1.8-3.3X over monomer for inbred mice and 2.3-3.4X over monomer in outbred mice. These showcase the biased CTL priming ability of our designed E7 nanoparticle constructs, key to potent vaccine responses.

### T-cell responses induced by designed E6 nanoparticles

We next determined the immunogenicity of our designed E6 nanoparticles as compared to monomer following the immunization scheme in **Figure 4A**. The groups assessed were E6_FL_Sol_Δp53_nano, E6_Tr_Sol_Δp53_nano, and the E6/E7 cocktail group to determine if there would be E6-directed synergy. These were compared to E6_FL_Δp53_monomer.

**Figure 5:**
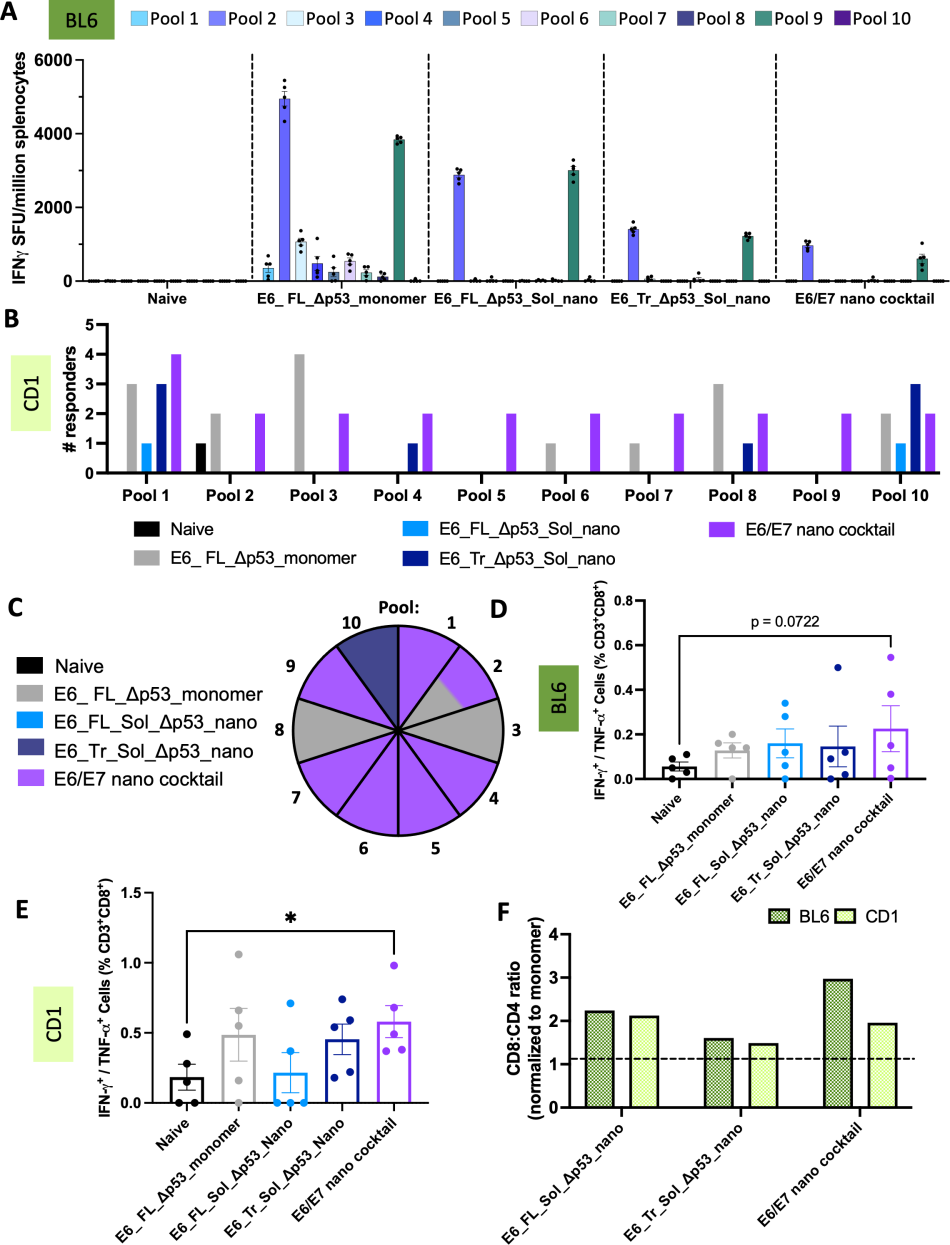
T-cell responses of designed E6 nanoparticles. **(A)** ELISpot responses in C57/BL6 mice. Splenocytes from immunized mice were stimulated with overlapping peptide pools that span WT E6. IFNγ spot-forming units per million splenocytes are quantified. (n=5 mice/group). Mean ± SEM; **(B)** Positive T-cell responders in CD-1 mice. ELISpot responses were quantified as above, with additional normalization to the average positive SFUs in the naïve group. Any mouse retaining strong responses after normalization to naïve was counted as a responder. The number of responders per pool per immunization group was determined. (n=5 mice/group). Black = naïve, gray = E6_FL_Δp53_monomer, light blue = E6_FL_Sol_Δp53_nano, dark blue = E6_Tr_Sol_Δp53_nano, purple = E6/E7 nano cocktail; **(C)** Top immunogenic constructs per pool in CD-1 mice. The pool number is indicated per wedge, and colored according to the immunization group that had the highest number of mice responding for that pool. In cases where multiple constructs had the same number of responders, the wedge color is split accordingly. Color scheme is identical to **(B, D)** IFNγ^+^ or TNFα^+^ CD8^+^ T-cell responses in C57/BL6 mice. Splenocytes were stimulated with E6 peptides and protein transport inhibitors, and CD8^+^ T-cell responses were quantified by intracellular cytokine staining and flow cytometry. Mean ± SEM shown. One sided T-tests were conducted; **(E)** IFNγ^+^ or TNFα^+^ CD8^+^ T-cell responses in CD-1 mice. Splenocytes were analyzed as above. Mean ± SEM shown. One sided T-tests were conducted. *p<0.05; **(F)** Nanoparticle T-cell bias. CD8^+^:CD4^+^ T-cell ratios were determined as the average of (%)IFNγ^+^ or TNFα^+^ CD8^+^ T-cells/(%)IFNγ^+^ or TNFα^+^ CD4^+^ T-cells per group. These were normalized to the average CD8^+^:CD4^+^ ratio for the E6 monomer to determine the fold CD8^+^:CD4^+^ stimulation bias for nanoparticle groups over monomer. Anything above the dotted line is superior to monomer.

Unexpectedly, the E6_FL_Δp53 monomer had the highest responses in inbred mice via ELISpot, though all nanoparticle groups also were able to elicit E6-directed responses (**Figure 5A**). The monomer does not contain Sol mutations which perhaps contributes to the differences observed. Overall, the strongest responses across all groups are to pool 2 and pool 9 in the genetically identical BL6 mice background. These pools overlap with the MHC D^b^-restricted E6 peptide YRDGNPYAV (48).

To better compare the ability of our nanoparticles to elicit diverse immune responses, we also assessed the E6 groups in an outbred CD-1 mouse mode. We observed responses to a much greater variety of pools in the outbred mice for both the monomer and nanoparticle groups (**Supplementary Figure S8B** **).** For more effective comparisons, we again characterized the number of positive responders in each group against each E6 pool; this demonstrates the diversity of responses (**Figure 5B**).

Akin to E7, the E6 group that elicited the highest number of responders on a per-pool basis was determined (**Figure 5C**). The combination E6/E7 nano cocktail group clearly elicits the highest number of responders across pools, with the most responders in pools 1, 4-7, and 9, and a tie for pool 2. This suggests that the presence of E7-directed nanoparticles in the cocktail may help cross-prime a stronger E6-directed response as well.

Though the strongest ELISpot responses for inbred BL6 mice were detected in the monomer group, it is interesting that the monomer does not have the highest average IFNγ^+^ or TNFα^+^ CD8^+^ T-cells for BL6 mice (**Figure 5D**). In fact, though the cocktail group had the weakest ELISpot responses, this group had the highest IFNγ^+^ or TNFα^+^ CD8^+^ responses for BL6 mice. For outbred CD-1 mice, the cocktail group similarly had the highest IFNγ^+^ or TNFα^+^ CD8^+^ responses (**Figure 5E**); in this case, the trend matches with the highest number of responders detected for the cocktail group. One possible reason for this phenomenon could be that the monomer elicits a more CD4^+^-biased response. The monomer has the lowest CD8^+^:CD4^+^ T-cell ratio for both BL6 and CD-1 mice out of all groups (**Supplementary Figure S8D**) suggesting weaker elicitation of CTLs; strong ELISpot responses may be due solely to T-cell help CD4^+^ responses.

All of the nanoparticle groups elicit stronger CD8^+^ biased responses than the monomer, ranging from 1.6-2.2X or 1.5-2.1X more CD8^+^ bias than monomer for BL6 and CD-1 mice, respectively (**Figure 5F**). All told, we see more potent CTL-like responses across all designed nanoparticle groups, and in a genetically diverse model more reflective of population MHC diversity, we observe the highest degree of responses in the E6/E7 cocktail group.

### Expanded epitope targeting of nanoparticle vaccines

**Figure 6:**
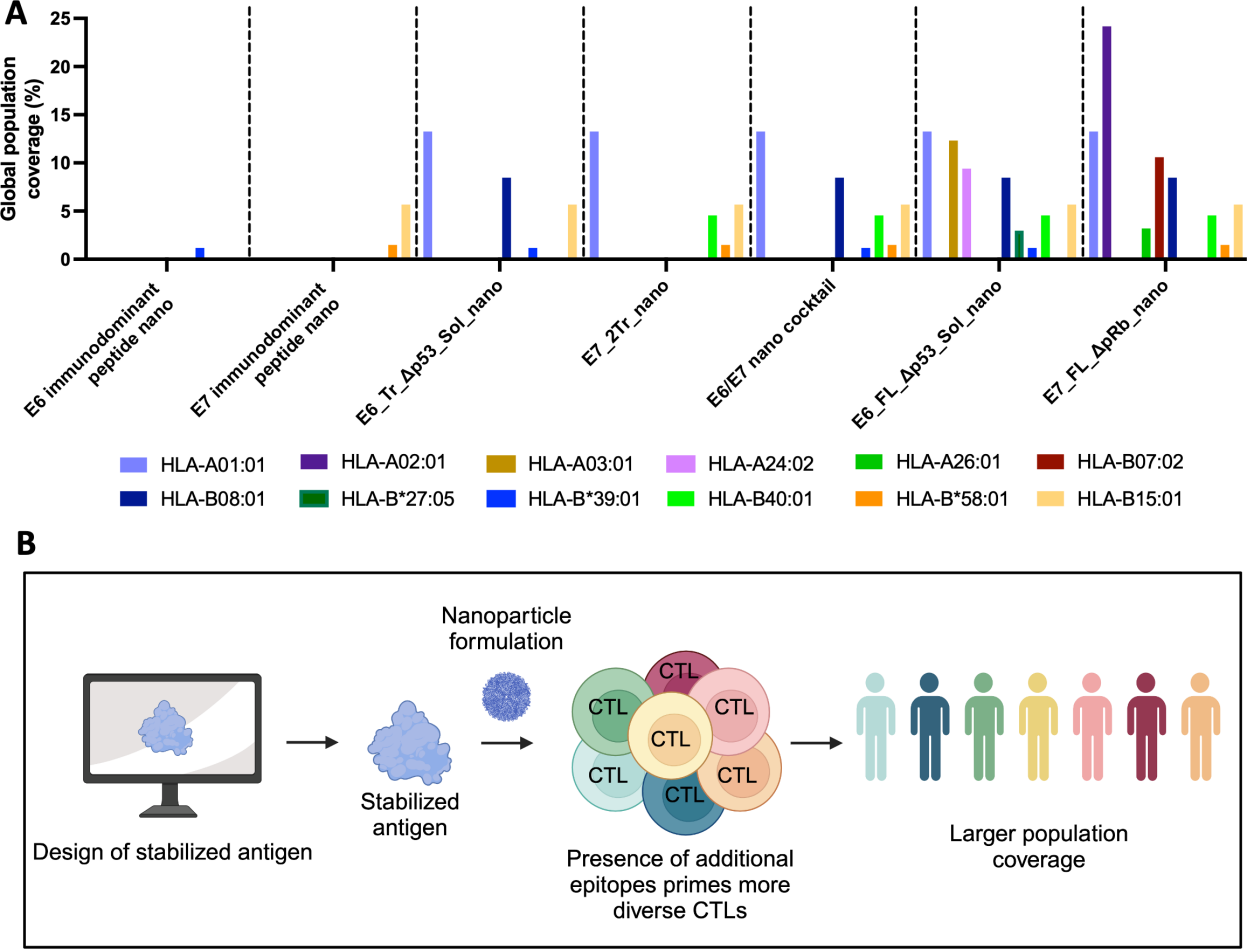
Expanded epitope targeting of nanoparticle vaccines. **(A)** Predicted binding of common human alleles to E6 or E7 epitopes from the indicated nanoparticles. Peptides predicted to have strong binding to each supertype representative allele were predicted using NetMHCPan for both E7 and E6. If the epitope for the peptide was present in the indicated nanoparticle, then the allele is graphed by its frequency in the global human population. E7 nanoparticles were predicted against E7_FL_ΔpRb; E6 nanoparticles were predicted against E6_FL_Δp53; the cocktail was predicted against both; **(B)** Overview of expanded epitope nanoparticle vaccine workflow. This approach uses computational tools to design stabilized, more full-length cancer antigens scaffolded onto nanoparticles with the aim of providing maximal immunogenic epitopes to the immune system. This allows for a greater diversity of T-cell responses and subsequently should allow a greater portion of the population to generate CTL responses to the vaccine.

We wanted to determine how the designed nanoparticles might translate to humans. We have demonstrated that the nanoparticles can target expanded epitopes in a mouse system, but mouse and human MHC alleles present peptides differently. To determine how our designed nanoparticles might function, we used NetMHCPan (49) to predict which E6 and E7 peptides would bind different HLA alleles in humans (**Figure 6A**; **Supplementary Figure S9**). We used representative HLA supertype alleles to determine how some of the more common HLA alleles would bind peptides and compared these predicted epitopes against the sequences of our designed nanoparticles. Because we are also interested in creating a vaccine targeted not just for the United States, but for a global population, we also graphed the frequency at which a given allele is present in the worldwide population.

Previous DNA-launched cancer-targeting nanoparticles have only scaffolded peptides (28, 30). In contrast, our designed, more full-length DNA-launched nanoparticles cover more alleles and a greater portion of the global population than a corresponding E7 or E6 peptide-only approach. Specifically, both E7_FL_ΔpRb_nano and E7_2Tr_nano afford greater coverage than an E7 peptide-only approach, and both E6_FL_Sol_Δp53_nano and E6_Tr_Sol_Δp53_nano afford more coverage than an E6 peptide-only. In addition, the E6/E7 cocktail can elicit immunity in a greater number of alleles than either E7_2Tr_nano or E6_Tr_Sol_Δp53_nano alone. While both full-length nanoparticles (E7_FL_ΔpRb_nano and E6_FL_Sol_Δp53_nano) have the largest theoretical population coverage, it is important to note that maximum theoretical coverage can only be achieved if the vaccine is in fact immunogenic. In our *in vivo* immunogenicity experiments, in the CD-1 heterogeneous mouse model to mimic human population diversity, both full-length nanoparticle constructs did not perform as strongly as the designed nanoparticle cocktail, supporting the importance of these designed immunogens in next-generation vaccines. Thus, the ideal vaccine candidate will weigh determined immunogenicity with theoretical population coverage; in this case, the E6/E7 nano cocktail achieved a strong balance of both.

Given the results explored within this paper, we propose the following schematic of how expanded epitope nanoparticle vaccines could be designed for alternate immunogens (**Figure 6B**). Computational design aids in the selection of lead stabilized antigen vaccine candidates. Formulation of stabilized antigens as DNA-launched nanoparticles results in the priming of multiple T-cell epitopes, which can help reduce MHC class restriction and afford greater population coverage. The application of this workflow to HPV16 E6 and E7 antigens resulted in the development of a non-oncogenic, multi-epitope targeting vaccine candidate.

## Discussion

Here, we described a generalizable workflow to create stabilized, more full-length nanoparticle vaccines and used it to design HPV16 E7 and E6 nanoparticle vaccine immunogens. This includes designed versions with oncogenic properties removed for the HPV16 E7 and E6 antigens, thus making them more translational. The use of AI structural prediction algorithms as well as computational tools increase the power and novelty of this design platform. We harness these and demonstrate that designs created using AI-driven techniques successfully form *in vitro* and retain their structure (**Figures 2**, **3**; **Supplementary Figures S4**, **S5**). *In vivo* immunogenicity experiments in both inbred and a more translationally relevant outbred mouse model showed that designed nanoparticles were immunogenic and capable of eliciting strong CD8+ T-cell responses (**Figures 4**, **5**; **Supplementary Figure S8**). In particular, designed nanoparticles elicited a strongly biased CD8+ T-cell response over monomer (**Figure 4G**; **Figure 5F**). The combination immunization group of both E6 and E7 nanoparticles, the E6/E7 nano cocktail, was able to induce the most positive responders in outbred mouse models and likewise would be predicted to elicit immunogenicity for several common HLA alleles (**Figures 4C, D**, **5B, C**, **6A**).

We chose to focus on disulfide engineering as a means to stabilize designs since it is a covalent modification in which minimal mutations can achieve the desired results. This may limit the potential impact on immunogenicity. Stabilization of a wide variety of proteins has been reported with only a single engineered disulfide bond (43–45). While other stabilization options exist, methods like proline substitution, or protein core or surface optimization may require multiple mutations to achieve the desired effect (41, 42, 50). However, these methods, in addition to non-covalent salt bridge engineering, warrant further exploration. It will be critical to preserve the WT sequence as much as possible when designing stabilized constructs, no matter the method chosen.

One question this study brings up is the link between structure, expression, and immunogenicity. We were unable to form and purify monomers, and E7_FL_ΔpRb_nano had lower expression levels, in concordance with its partially unfolded structural prediction. In general, both E7 and E6 full-length nanoparticles and their derivatives generally had poorer expression *in vitro* than their corresponding truncated antigen counterparts. However, despite this poorer *in vitro* expression*, in vivo* E7_FL_ΔpRb_nano elicited the strongest response for E7, and E6_FL_Δp53_monomer elicited the strongest ELISpot response for E6 in BL6 mice. One possible explanation for these findings could be that an unfolded antigen might be advantageous for more efficient MHC-I presentation. Proteins that don’t achieve native folds will be sent for degradation; these degraded proteins are a huge source of peptides that are eventually loaded onto MHC-I for T-cell surveillance (51, 52). If, for example, the E6 monomer and E7_FL_ΔpRb_nano express poorly and are often misfolded, they may be frequently targeted for degradation and end up being presented on MHC-I more often. Indeed, there has been some suggestion that a different HPV antigen, E1, which is unstructured, could make an attractive new vaccine target for this very reason (53).

However, at odds with this idea is that E7_2Tr_nano and the E6/E7 cocktail elicited stronger immunity than the E7 monomer, despite expressing far better *in vitro* and presumably having much higher stability. Further, in CD-1 mice, the E6/E7 cocktail composed of two truncated nanoparticles, E7_2Tr_nano and E6_Tr_Sol_Δp53_nano, outperformed monomer and full-length nanoparticles for both E7 and E6. Dendritic cells may play a large role in eliciting this stronger immunity in DNA-delivery platforms. Antigens that have been properly assembled and secreted, such as the presumably well-structured truncated nanoparticles, can be taken up by antigen-presenting cells such as dendritic cells for cross-presentation on MHC-I (54–56), thereby increasing overall immunogenicity. Therefore, the overall ability to prime immunogenicity may be driven by a balance of both degraded antigens and well-folded secreted antigens. Finding a good balance, if one exists, may inform future vaccine design.

One critical consideration for full-length nanoparticle vaccine design is also a balance of the actual ability to prime immunity balanced with the theoretical ability to have more epitopes recognized by a global suite of HLA molecules. While full-length nanoparticles contain more theoretical epitopes, as we observed in the more translationally relevant CD-1 mouse model, they were inferior at eliciting immunity in comparison to their truncated, stabilized counterparts. Thus, vaccine decisions need to balance these possibilities or weigh allele frequencies in target populations. In cases where there are known human CTL epitopes, these could also be appended via flexible linkers to the stabilized truncated domain, possibly allowing a balance between good immunogenicity and higher theoretical coverage.

There are numerous HPV-targeting vaccines in clinical trials (20) focused on improving T-cell responses in order to drive viral clearance. Many of these vaccines use monomeric versions of E6 or E7 to drive an immune response (22, 25, 27). Here we demonstrate that our nanoparticles elicit stronger CD8^+^ biased responses over monomer, offering one potential way to further improve clinical responses. Other vaccines in clinical trials use alternate CD8+ enhancing strategies, like the incorporation of HSP70 to promote antigen processing and presentation (27, 57). Combining these or other strategies to augment CD8+ T-cell responses could help boost immunogenicity.

All told, these findings offer both a starting point for the development of vaccines with improved T-cell generation and suggest applications that could include HPV therapeutic vaccine approaches as described. The computationally driven E6 and E7 nanoparticle designs incorporate mutations to mitigate the risk of oncogenesis and drive T-cell responses to multiple E6 and E7 epitopes. Relevant future efforts should assess the elicitation of T-cell responses in additional model systems. Generally, the incorporation of design strategies as we present here will be important for the development of improved immunotherapeutic vaccines with translational relevance.

## Materials and methods

### Sequences

All starting sequences used for both E6 and E7 were from the HPV16 subtype. The full-length WT sequence for E7 was obtained from UniProt (Accession number: P03129). Any ΔprB constructs contained mutations and deletions in regions that bind pRb as previously described (46).

The full-length WT sequence for E6 was obtained from UniProt (Accession number: P03126). Any constructs designated with ‘Sol’ were based on PDB:6SJA and contained mutations that interrupt the E6 homodimerization domain (F47R) and prevent disulfide-mediated aggregation (4C/4S) as previously described (58, 59). Any Δp53 constructs contained mutations and deletions in regions that bind p53 as previously described (46).

### Structural prediction

Full-length antigen structures were predicted using either AlphaFold2 only (E6) or AlphaFold2 and RoseTTAFold2 (E7). In all cases, five structures were predicted per input sequence. AlphaFold structures were predicted using non-templated ColabFold, an online accessible version of AlphaFold2 (31, 60). A local installation of RoseTTAFold2 (35) was downloaded from (https://github.com/RosettaCommons/RoseTTAFold) and also used to run predictions. All structures were loaded into PyMOL 2.0 for visualization. The top-ranked structure, or in some cases, structures with unique folds, was then used for further domain engineering. Per-residue pLDDT scores (AlphaFold) or RMS-error scores (RoseTTAFold) were also extracted from models of interest. A crystal structure of E6_FL_Sol with only an additional N-terminal glycine was available (PDB:6SJA), and so no structural prediction was needed for domains based on this full-length sequence unless they contained the additional Δp53 mutations.

### Domain minimization

Determination of purported foldable domains was determined through a variety of methods. This included the use of pLDDT/RMS-error. For example, pLDDT scores generally predict stable domains and regions of disorder quite well (37, 38, 61). Thus, pLDDT scores can be used to determine regions of disorder. These per residue scores were used to down-select to structured regions of interest, which was then accompanied by visual inspection in Pymol of the new domains. Some partial structures of E7 for a different subtype (HPV45) were available in the PDB (2F8B, 2EWL) and were aligned to the AlphaFold2 and RosettaFold2 predictions to refine domain minimization. Once sequences of minimized domains were selected, the structures were re-predicted using Alphafold2 to ensure that the truncations were predicted to maintain the desired geometries.

After initial rounds of characterization of minimized designs, the most successful E6 minimizations were engineered to incorporate the Δp53 mutations, and E7 minimizations were engineered to incorporate the ΔpRb mutations.

### Disulfide scanning

For additional stability, the engineering of disulfide bonds to stabilize local protein folds was considered. Using an MSL library (62) a disulfide scanning tool called FindDisulfides was created. It takes an input PDB file of the protein to be designed, then scans for candidate pairs of residues to create a disulfide bridge. The backbone geometries of each pair of residues are compared against the PDB to determine how many other structures in the PDB contain disulfide pairs with similar geometries. New disulfide bridges under consideration were then filtered so that mutated residues had to be >20 residues apart and have >500 matching PDB geometries. The disulfides that met these criteria were used to model disulfides in the input starting structure. Purported disulfide bridges were manually inspected in PyMOL and combinations of disulfides were determined from there (1-2 disulfide pairs per structure). If the structure contained more than one pair of disulfides, efforts were made to have them located on distal sides in order to avoid the mispairing of cysteines.

### Nanoparticle design

Any construct with the designation ‘nano’ was scaffolded onto a stabilized, engineered lumazine synthase scaffold (previously described as DLnano_LS_GT8) (28). All constructs were cloned into the pVax vector and contained the IgE leader sequence. Constructs were codon optimized for *homo sapiens* and/or *mus musculus*; all E6 and E7 nanoparticle constructs and their derivatives were codon optimized identically to the monomer.

### Nanoparticle production and characterization

Each nanoparticle was transfected into ExpiF293 cells following the manufacturer’s guidelines ((ExpiFectamine™ 293 Transfection Kit(Gibco)) with a transfection size of 100mL. Supernatants were harvested 7 days post-transfection and purified using an in-house column packed with Galnthus Nivalis Lectin Beads (Vector Lab) on an AKTA Pure system. Following lectin purification, fractions were pooled, concentrated, and buffer exchanged into 1X PBS. Size exclusion chromatography (SEC) was then run using a Superose 6 10/300 GL Increase column (Cytiva), again using an AKTA Pure system. In some cases, two rounds of size exclusion chromatography were run. The relevant fractions were then pooled and concentrated. Concentrations of the nanoparticles were then determined using a NanoDrop ™ One spectrophotometer (Thermo Scientific) to calculate the final transfection yields of the purified nanoparticles.

### ELISAs

All ELISAs were performed using polystyrene high binding, 96-well Flat-Bottom, Half-Area Microplates (Corning). Plates were coated at 15μg/mL with the relevant E6 or E7 nanoparticle overnight at 4°C, washed with 1X PBS/0.05% Tween-20, then blocked for 1hr at RT with 5% milk/1X PBS/0.01% Tween-20. Following the wash, dilutions of the relevant antibody were performed. For E6 ELISAs, an anti-E6 monoclonal antibody (MAB874, Millipore Sigma) was prepared in duplicate (either 50μg/mL or 70μg/mL starting concentration, 3X dilution series). For E7 ELISAs, an anti-E7 polyclonal antibody(PA5-117383, Fisher Scientific) was prepared in duplicate (70μg/mL starting concentration, 3X dilution series). Plates were then incubated for 1 hour at RT, washed, then detected for 45min at RT with 1:10,000 of the relevant secondary antibody. For E6 ELISAs, anti-mouse H+L-HRP (Bethyl, A90-116P) was used and for E7 ELISAs, anti-rabbit H+L-HRP was used (Bethyl, A120-201P). Following the wash, plates were incubated with 1-StepTM Ultra TMB-ELISA Substrate Solution (Thermo Scientific) for 1 min before being quenched with 1 M H_2_SO_4_. The absorbance of plates was then read at 450nm and 570nm using a Biotek Synergy 2 plate reader. Absorbance was 450nm-570nm normalized and the background of blank wells was subtracted. All data was exported to Microsoft Excel and analyzed in GraphPad Prism 10.

### Negative stain electron microscopy

Purified nanoparticles in PBS (3uL) at 0.03-0.05 mg/mL were adsorbed onto glow-discharged carbon-coated Cu400 EM grids. Grids were rinsed several times with TBS. The grids were then stained with 3 µL of 2% uranyl formate, immediately blotted, and stained again with 3 µL of the stain for 90 seconds, followed by a final blot. A FEI Tecnai T12 microscope equipped with a Oneview Gatan camera at 52000× magnification was used for data collection. Data is at a 2.356 Å/pixel ratio.

### Animal use

All animal work was performed under protocols approved by The Wistar Institute Institutional Animal Care and Use Committee. Mice were housed in the Wistar Institute Animal Facility and given free access to food and water.

### Immunizations

Six to eight week old female C57BL/6J (The Jackson Laboratory) or CD-1 IGS mice (Charles River) were obtained. To obtain cellular responses, mice were immunized with 10μg of the relevant E6 or E7 DNA vaccine, or 20μg total in the case of combination groups, in their *tibialus anterior* muscle. To promote plasmid uptake use of a CELLECTRA EP(Inovio Pharmaceuticals) delivery device was employed. Two sets of 0.2 A pulses with a 3-second interval were delivered; each pulse consisted of 52 ms pulses with 198 ms delay. Mice received an identical vaccination two weeks post-initial immunization. At week 3, terminal bleeds were collected and mice were euthanized under CO_2_. Spleens were collected into RPMI media supplemented with 10% HI FBS and 1% P/S, then processed using a Seward Stomacher 80 (Seward) followed by filtration through 40 µm cell strainers. Red blood cells were lysed using ACK lysis buffer (Thermo Fisher Scientific).

### ELISpot

Cellular responses were quantified using ELISpot assays. Briefly, 200,000 splenocytes were plated onto mouse IFNγ ELI-SpotPLUS plates (MabTech) and stimulated with 5 µg/mL peptides. Overlapping peptide pools were constructed so that the length of E6 or E7 was spanned by overlapping peptides (15AA long, 8AA overlaps). This method of epitope determination has been previously described (63). Peptides spanning E6 were pooled into 10 different pools, while those spanning E7 were pooled into 8 different pools. Splenocytes from the relevant mice were incubated with each of the relevant peptide pools for 20 hours at 37°C, then developed in accordance with the manufacturer’s instructions. Concanavalin A or R10 were used as positive or negative controls, respectively. Spots were quantified using a MabTech IRIS Fluorospot/ELIspot reader, normalized to an unstimulated control (R10).

For additional normalization in CD-1 mice, ‘responders’ were defined from ELISpot data. The average of positive spots was determined for naïve mice, and this value was subtracted from all ELISpot data. If a mouse still had >300 positive spots post-normalization, it was counted as a positive responder to that pool. Some mice had such high responses the plate reader could not quantify the spots. While these mice were excluded in **Supplementary Figure S8** because no numeric value can be assigned as ‘above the limit of detection for responses’, they were included in the responders analysis as a positive responder.

### Intracellular staining and flow cytometry

Splenocytes (1M cells/well) were isolated as described in the *Immunizations* section, then stimulated with peptide pools for full-length E6 or E7 in the presence of protein transport inhibitor for 5 hours at 37°C. Cells were then stained with anti-mouse CD3-PE-Cy5, CD4-BV510, CD8-APC-Cy7, IFNγ-APC, IL-2-PE-Cy7 and TNFα-BV605. All antibodies were purchased from BioLegend. To assess cellular viability, cells were also stained with Live/Dead violet (Invitrogen). Samples were then run on an 18-color LSRII flow cytometer (BD Biosciences), gated relative to naïve mice, and analyzed by FlowJo software.

### HLA allele binding specificities and frequencies

Peptides predicted to bind human HLA alleles were determined using NetMHCpan (64). Due to the extreme diversity of the human HLA repertoire, predictions were generated using the ‘HLA supertype representative’ set of loci, which are HLA proteins clustered according to similar binding specificities (49, 65, 66). These consist of HLA-A*02:01, HLA-A*01:01, HLA-A*03:01, HLA-A*24:02, HLA-A*26:01, HLA-B*07:02, HLA-B*08:01, HLA-B*27:05, HLA-B*39:01, HLA-B*40:01, HLA-B*58:01, and HLA-B*15:01. Binding peptides (9-mers) were determined from E7_FL_ ΔpRb or E6_FL_Δp53 sequences for E7 and E6, respectively. The threshold for strong binders was specified as 0.5% rank.

Population frequencies of the HLA supertype alleles were determined using the HLA allele report from the Allele Frequency Net Database (67). Percentages were determined as 100*allele count/number of people typed for a given allele and are based on their gold datasets.

### Statistical analysis

Statistical analyses were performed using GraphPad Prism v10.0.3. For comparisons between individual groups, one-sided T-tests were conducted due to sample sizes of n=5.

## Data Availability

The original contributions presented in the study are included in the article/**Supplementary Material**. Further inquiries can be directed to the corresponding author.

## References

[1] de Martel C Plummer M Vignat J Franceschi S. Worldwide burden of cancer attributable to HPV by site, country and HPV type. Int J Cancer. (2017) 141:664–70. doi: 10.1002/ijc.v141.4 PMC5520228 28369882

[2] Humans IWGotEoCRt. Human papillomaviruses. IARC Monogr Eval Carcinog Risks Hum. (2007) 90:1–636. 18354839 PMC4781057

[3] Serrano B Brotons M Bosch FX Bruni L. Epidemiology and burden of HPV-related disease. Best Pract Res Clin Obstet Gynaecol. (2018) 47:14–26. doi: 10.1016/j.bpobgyn.2017.08.006 29037457

[4] Balhara N Yadav R Ranga S Ahuja P Tanwar M. Understanding the HPV associated cancers: A comprehensive review. Mol Biol Rep. (2024) 51:743. doi: 10.1007/s11033-024-09680-6 38874682

[5] Meites E Szilagyi PG Chesson HW Unger ER Romero JR Markowitz LE. Human papillomavirus vaccination for adults: updated recommendations of the advisory committee on immunization practices. MMWR Morb Mortal Wkly Rep. (2019) 68:698–702. doi: 10.15585/mmwr.mm6832a3 31415491 PMC6818701

[6] World Health Organization (WHO). Human papillomavirus vaccines: WHO position paper. Wkly Epidemiol Rec. (2022) 97:645–72. Available online at: https://www.who.int/publications/i/item/who-wer9750-645-672.

[7] Pal A Kundu R. Human papillomavirus E6 and E7: the cervical cancer hallmarks and targets for therapy. Front Microbiol. (2019) 10:3116. doi: 10.3389/fmicb.2019.03116 32038557 PMC6985034

[8] Harden ME Munger K. Human papillomavirus molecular biology. Mutat Res Rev Mutat Res. (2017) 772:3–12. doi: 10.1016/j.mrrev.2016.07.002 28528688 PMC5500221

[9] Aksoy P Gottschalk EY Meneses PI. HPV entry into cells. Mutat Res Rev Mutat Res. (2017) 772:13–22. doi: 10.1016/j.mrrev.2016.09.004 28528686 PMC5443120

[10] The Cancer Genome Atlas Research Network. Integrated genomic and molecular characterization of cervical cancer. Nature. (2017) 543:378–84. doi: 10.1038/nature21386 PMC5354998 28112728

[11] Jeon S Allen-Hoffmann BL Lambert PF. Integration of human papillomavirus type 16 into the human genome correlates with a selective growth advantage of cells. J Virol. (1995) 69:2989–97. doi: 10.1128/jvi.69.5.2989-2997.1995 PMC188998 7707525

[12] McBride AA Warburton A. The role of integration in oncogenic progression of HPV-associated cancers. PloS Pathog. (2017) 13:e1006211. doi: 10.1371/journal.ppat.1006211 28384274 PMC5383336

[13] Hoppe-Seyler K Bossler F Braun JA Herrmann AL Hoppe-Seyler F. The HPV E6/E7 oncogenes: key factors for viral carcinogenesis and therapeutic targets. Trends Microbiol. (2018) 26:158–68. doi: 10.1016/j.tim.2017.07.007 28823569

[14] Jeon S Lambert PF. Integration of human papillomavirus type 16 DNA into the human genome leads to increased stability of E6 and E7 mRNAs: implications for cervical carcinogenesis. Proc Natl Acad Sci U S A. (1995) 92:1654–8. doi: 10.1073/pnas.92.5.1654 PMC42578 7878034

[15] Scheffner M Huibregtse JM Vierstra RD Howley PM. The HPV-16 E6 and E6-AP complex functions as a ubiquitin-protein ligase in the ubiquitination of p53. Cell. (1993) 75:495–505. doi: 10.1016/0092-8674(93)90384-3 8221889

[16] Scheffner M Werness BA Huibregtse JM Levine AJ Howley PM. The E6 oncoprotein encoded by human papillomavirus types 16 and 18 promotes the degradation of p53. Cell. (1990) 63:1129–36. doi: 10.1016/0092-8674(90)90409-8 2175676

[17] Boyer SN Wazer DE Band V. E7 protein of human papilloma virus-16 induces degradation of retinoblastoma protein through the ubiquitin-proteasome pathway. Cancer Res. (1996) 56:4620–4. 8840974

[18] Mir BA Ahmad A Farooq N Priya MV Siddiqui AH Asif M. Increased expression of HPV-E7 oncoprotein correlates with a reduced level of pRb proteins *via* high viral load in cervical cancer. Sci Rep. (2023) 13:15075. doi: 10.1038/s41598-023-42022-3 37699974 PMC10497568

[19] Yang A Jeang J Cheng K Cheng T Yang B Wu TC. Current state in the development of candidate therapeutic HPV vaccines. Expert Rev Vaccines. (2016) 15:989–1007. doi: 10.1586/14760584.2016.1157477 26901118 PMC4977850

[20] Mo Y Ma J Zhang H Shen J Chen J Hong J. Prophylactic and therapeutic HPV vaccines: current scenario and perspectives. Front Cell Infect Microbiol. (2022) 12:909223. doi: 10.3389/fcimb.2022.909223 35860379 PMC9289603

[21] Massarelli E William W Johnson F Kies M Ferrarotto R Guo M. Combining immune checkpoint blockade and tumor-specific vaccine for patients with incurable human papillomavirus 16-related cancer: A phase 2 clinical trial. JAMA Oncol. (2019) 5:67–73. doi: 10.1001/jamaoncol.2018.4051 30267032 PMC6439768

[22] Arribillaga L Echeverria I Belsue V Gomez T Lozano T Casares N. Bivalent therapeutic vaccine against HPV16/18 genotypes consisting of a fusion protein between the extra domain A from human fibronectin and HPV16/18 E7 viral antigens. J Immunother Cancer. (2020) 8(1):e000704. doi: 10.1136/jitc-2020-000704 32581060 PMC7319778

[23] Choi YJ Hur SY Kim TJ Hong SR Lee JK Cho CH. Prospective, randomized, multicenter, open-label study of GX-188E, an HPV DNA vaccine, in patients with cervical intraepithelial neoplasia 3. Clin Cancer Res. (2020) 26:1616–23. doi: 10.1158/1078-0432.CCR-19-1513 31727676

[24] Alvarez RD Huh WK Bae S Lamb LS Jr. Conner MG Boyer J. A pilot study of pNGVL4a-CRT/E7(detox) for the treatment of patients with HPV16+ cervical intraepithelial neoplasia 2/3 (CIN2/3). Gynecol Oncol. (2016) 140:245–52. doi: 10.1016/j.ygyno.2015.11.026 PMC4724445 26616223

[25] Trimble CL Morrow MP Kraynyak KA Shen X Dallas M Yan J. Safety, efficacy, and immunogenicity of VGX-3100, a therapeutic synthetic DNA vaccine targeting human papillomavirus 16 and 18 E6 and E7 proteins for cervical intraepithelial neoplasia 2/3: a randomised, double-blind, placebo-controlled phase 2b trial. Lancet. (2015) 386:2078–88. doi: 10.1016/S0140-6736(15)00239-1 PMC4888059 26386540

[26] INOVIO Announces Positive Results from REVEAL 1, a Phase 3 Pivotal Trial Evaluating VGX-3100, its DNA- based HPV Immunotherapy for the Treatment of High-grade Precancerous Cervical Dysplasia Caused by HPV-16 and/or HPV-18. INOVIO Phamaceuticals, Inc. (2021). Available online at: https://ir.inovio.com/news-releases/news-releases-details/2021/INOVIO-Announces-Positive-Results-from-REVEAL-1-a-Phase-3-Pivotal-Trial-Evaluating-VGX-3100-its-DNA-based-HPV-Immunotherapy-for-the-Treatment-of-High-grade-Precancerous-Cervical-Dysplasia-Caused-by-HPV-16-andor-HPV-18/default.aspx.

[27] Einstein MH Roden RBS Ferrall L Akin M Blomer A Wu TC. Safety run-in of intramuscular pNGVL4a-sig/E7(detox)/HSP70 DNA and TA-CIN protein vaccination as treatment for HPV16+ ASC-US, ASC-H, or LSIL/CIN1. Cancer Prev Res (Phila). (2023) 16:219–27. doi: 10.1158/1940-6207.CAPR-22-0413 PMC10068439 36607735

[28] Xu Z Chokkalingam N Tello-Ruiz E Wise MC Bah MA Walker S. A DNA-launched nanoparticle vaccine elicits CD8(+) T-cell immunity to promote *in vivo* tumor control. Cancer Immunol Res. (2020) 8:1354–64. doi: 10.1158/2326-6066.CIR-20-0061 PMC7642117 32913042

[29] Xu Z Wise MC Chokkalingam N Walker S Tello-Ruiz E Elliott STC. *In vivo* assembly of nanoparticles achieved through synergy of structure-based protein engineering and synthetic DNA generates enhanced adaptive immunity. Adv Sci. (2020) 7:1902802. doi: 10.1002/advs.201902802 PMC7175333 32328416

[30] Tursi NJ Xu Z Helble M Walker S Liaw K Chokkalingam N. Engineered antibody cytokine chimera synergizes with DNA-launched nanoparticle vaccines to potentiate melanoma suppression *in vivo* . Front Immunol. (2023) 14:1072810. doi: 10.3389/fimmu.2023.1072810 36911698 PMC9997082

[31] Jumper J Evans R Pritzel A Green T Figurnov M Ronneberger O. Highly accurate protein structure prediction with AlphaFold. Nature. (2021) 596:583–9. doi: 10.1038/s41586-021-03819-2 PMC8371605 34265844

[32] Wentzensen N Lahrmann B Clarke MA Kinney W Tokugawa D Poitras N. Accuracy and efficiency of deep-learning-based automation of dual stain cytology in cervical cancer screening. J Natl Cancer Inst. (2021) 113:72–9. doi: 10.1093/jnci/djaa066 PMC7781458 32584382

[33] Stillman NR Balaz I Tsompanas M-A Kovacevic M Azimi S Lafond S. Evolutionary computational platform for the automatic discovery of nanocarriers for cancer treatment. NPJ Comput Materials. (2021) 7(1):150. doi: 10.1038/s41524-021-00614-5

[34] MacMillan P Syed AM Kingston BR Ngai J Sindhwani S Lin ZP. Toward predicting nanoparticle distribution in heterogeneous tumor tissues. Nano Lett. (2023) 23:7197–205. doi: 10.1021/acs.nanolett.3c02186 37506224

[35] Baek M DiMaio F Anishchenko I Dauparas J Ovchinnikov S Lee GR. Accurate prediction of protein structures and interactions using a three-track neural network. Science. (2021) 373:871–6. doi: 10.1126/science.abj8754 PMC7612213 34282049

[36] Abramson J Adler J Dunger J Evans R Green T Pritzel A. Accurate structure prediction of biomolecular interactions with AlphaFold 3. Nature. (2024) 630:493–500. doi: 10.1038/s41586-024-07487-w 38718835 PMC11168924

[37] Piovesan D Monzon AM Tosatto SCE. Intrinsic protein disorder and conditional folding in AlphaFoldDB. Protein Sci. (2022) 31:e4466. doi: 10.1002/pro.v31.11 36210722 PMC9601767

[38] Tunyasuvunakool K Adler J Wu Z Green T Zielinski M Zidek A. Highly accurate protein structure prediction for the human proteome. Nature. (2021) 596:590–6. doi: 10.1038/s41586-021-03828-1 PMC8387240 34293799

[39] Dauparas J Anishchenko I Bennett N Bai H Ragotte RJ Milles LF. Robust deep learning-based protein sequence design using ProteinMPNN. Science. (2022) 378:49–56. doi: 10.1126/science.add2187 36108050 PMC9997061

[40] Guo H-B Perminov A Bekele S Kedziora G Farajollahi S Varaljay V. AlphaFold2 models indicate that protein sequence determines both structure and dynamics. Sci Rep. (2022) 12(1):10696. doi: 10.1038/s41598-022-14382-9 35739160 PMC9226352

[41] Hsieh CL Goldsmith JA Schaub JM DiVenere AM Kuo HC Javanmardi K. Structure-based design of prefusion-stabilized SARS-CoV-2 spikes. Science. (2020) 369:1501–5. doi: 10.1126/science.abd0826 PMC7402631 32703906

[42] Leonard AC Weinstein JJ Steiner PJ Erbse AH Fleishman SJ Whitehead TA. Stabilization of the SARS-CoV-2 receptor binding domain by protein core redesign and deep mutational scanning. Protein Eng Des Sel. (2022) 35:gzac002. doi: 10.1093/protein/gzac002 35325236 PMC9077414

[43] Jo BH Park TY Park HJ Yeon YJ Yoo YJ Cha HJ. Engineering *de novo* disulfide bond in bacterial alpha-type carbonic anhydrase for thermostable carbon sequestration. Sci Rep. (2016) 6:29322. doi: 10.1038/srep29322 27385052 PMC4935852

[44] Le QA Joo JC Yoo YJ Kim YH. Development of thermostable Candida Antarctica lipase B through novel in silico design of disulfide bridge. Biotechnol Bioeng. (2012) 109:867–76. doi: 10.1002/bit.v109.4 22095554

[45] Mansfeld J Vriend G Dijkstra BW Veltman OR Van den Burg B Venema G. Extreme stabilization of a thermolysin-like protease by an engineered disulfide bond. J Biol Chem. (1997) 272:11152–6. doi: 10.1074/jbc.272.17.11152 9111013

[46] Yan J Reichenbach DK Corbitt N Hokey DA Ramanathan MP McKinney KA. Induction of antitumor immunity *in vivo* following delivery of a novel HPV-16 DNA vaccine encoding an E6/E7 fusion antigen. Vaccine. (2009) 27:431–40. doi: 10.1016/j.vaccine.2008.10.078 PMC4477831 19022315

[47] Bagarazzi ML Yan J Morrow MP Shen X Parker RL Lee JC. Immunotherapy against HPV16/18 generates potent TH1 and cytotoxic cellular immune responses. Sci Transl Med. (2012) 4:155ra38. doi: 10.1126/scitranslmed.3004414 PMC4317299 23052295

[48] Feltkamp MC Smits HL Vierboom MP Minnaar RP de Jongh BM Drijfhout JW. Vaccination with cytotoxic T lymphocyte epitope-containing peptide protects against a tumor induced by human papillomavirus type 16-transformed cells. Eur J Immunol. (1993) 23:2242–9. doi: 10.1002/eji.1830230929 7690326

[49] Nielsen M Lundegaard C Blicher T Lamberth K Harndahl M Justesen S. NetMHCpan, a method for quantitative predictions of peptide binding to any HLA-A and -B locus protein of known sequence. PloS One. (2007) 2:e796. doi: 10.1371/journal.pone.0000796 17726526 PMC1949492

[50] Goverde CA Pacesa M Goldbach N Dornfeld LJ Balbi PEM Georgeon S. Computational design of soluble and functional membrane protein analogues. Nature. (2024) 631:449–58. doi: 10.1038/s41586-024-07601-y PMC11236705 38898281

[51] Reits EA Vos JC Gromme M Neefjes J. The major substrates for TAP *in vivo* are derived from newly synthesized proteins. Nature. (2000) 404:774–8. doi: 10.1038/35008103 10783892

[52] Schubert U Anton LC Gibbs J Norbury CC Yewdell JW Bennink JR. Rapid degradation of a large fraction of newly synthesized proteins by proteasomes. Nature. (2000) 404:770–4. doi: 10.1038/35008096 10783891

[53] Boilesen DR Nielsen KN Holst PJ. Novel antigenic targets of HPV therapeutic vaccines. Vaccines (Basel). (2021) 9(11):1262. doi: 10.3390/vaccines9111262 34835193 PMC8621534

[54] Kutzler MA Weiner DB. DNA vaccines: ready for prime time? Nat Rev Genet. (2008) 9:776–88. doi: 10.1038/nrg2432 PMC4317294 18781156

[55] Gary EN Weiner DB. DNA vaccines: prime time is now. Curr Opin Immunol. (2020) 65:21–7. doi: 10.1016/j.coi.2020.01.006 PMC7195337 32259744

[56] Pandya A Shah Y Kothari N Postwala H Shah A Parekh P. The future of cancer immunotherapy: DNA vaccines leading the way. Med Oncol. (2023) 40:200. doi: 10.1007/s12032-023-02060-3 37294501 PMC10251337

[57] Trimble CL Peng S Kos F Gravitt P Viscidi R Sugar E. A phase I trial of a human papillomavirus DNA vaccine for HPV16+ cervical intraepithelial neoplasia 2/3. Clin Cancer Res. (2009) 15:361–7. doi: 10.1158/1078-0432.CCR-08-1725 PMC2865676 19118066

[58] Nomine Y Masson M Charbonnier S Zanier K Ristriani T Deryckere F. Structural and functional analysis of E6 oncoprotein: insights in the molecular pathways of human papillomavirus-mediated pathogenesis. Mol Cell. (2006) 21:665–78. doi: 10.1016/j.molcel.2006.01.024 16507364

[59] Zanier K ould M'hamed ould Sidi A Boulade-Ladame C Rybin V Chappelle A Atkinson A. Solution structure analysis of the HPV16 E6 oncoprotein reveals a self-association mechanism required for E6-mediated degradation of p53. Structure. (2012) 20:604–17. doi: 10.1016/j.str.2012.02.001 PMC3325491 22483108

[60] Mirdita M Schutze K Moriwaki Y Heo L Ovchinnikov S Steinegger M. ColabFold: making protein folding accessible to all. Nat Methods. (2022) 19:679–82. doi: 10.1038/s41592-022-01488-1 PMC9184281 35637307

[61] Necci M Piovesan D Predictors C DisProt C Tosatto SCE. Critical assessment of protein intrinsic disorder prediction. Nat Methods. (2021) 18:472–81. doi: 10.1038/s41592-021-01117-3 PMC8105172 33875885

[62] Kulp DW Subramaniam S Donald JE Hannigan BT Mueller BK Grigoryan G. Structural informatics, modeling, and design with an open-source Molecular Software Library (MSL). J Comput Chem. (2012) 33:1645–61. doi: 10.1002/jcc.v33.20 PMC3432414 22565567

[63] Fiore-Gartland A Manso BA Friedrich DP Gabriel EE Finak G Moodie Z. Pooled-peptide epitope mapping strategies are efficient and highly sensitive: an evaluation of methods for identifying human T cell epitope specificities in large-scale HIV vaccine efficacy trials. PloS One. (2016) 11:e0147812. doi: 10.1371/journal.pone.0147812 26863315 PMC4749288

[64] Reynisson B Alvarez B Paul S Peters B Nielsen M. NetMHCpan-4.1 and NetMHCIIpan-4.0: improved predictions of MHC antigen presentation by concurrent motif deconvolution and integration of MS MHC eluted ligand data. Nucleic Acids Res. (2020) 48:W449–W54. doi: 10.1093/nar/gkaa379 PMC7319546 32406916

[65] Lund O Nielsen M Kesmir C Petersen AG Lundegaard C Worning P. Definition of supertypes for HLA molecules using clustering of specificity matrices. Immunogenetics. (2004) 55:797–810. doi: 10.1007/s00251-004-0647-4 14963618

[66] Sette A Sidney J. Nine major HLA class I supertypes account for the vast preponderance of HLA-A and -B polymorphism. Immunogenetics. (1999) 50:201–12. doi: 10.1007/s002510050594 10602880

[67] Gonzalez-Galarza FF McCabe A Santos E Jones J Takeshita L Ortega-Rivera ND. Allele frequency net database (AFND) 2020 update: gold-standard data classification, open access genotype data and new query tools. Nucleic Acids Res. (2020) 48:D783–D8. doi: 10.1093/nar/gkz1029 PMC7145554 31722398

