# Supplementary material for: Structural engineering of stabilized, expanded epitope nanoparticle vaccines for HPV
Source: Front Immunol. 2025 Jan 31;16:1535261. doi: 10.3389/fimmu.2025.1535261 (PMC11826081; doi:10.3389/fimmu.2025.1535261)
Supplement: Supplementary file 1 [file DataSheet1.pdf]

Supplementary Material

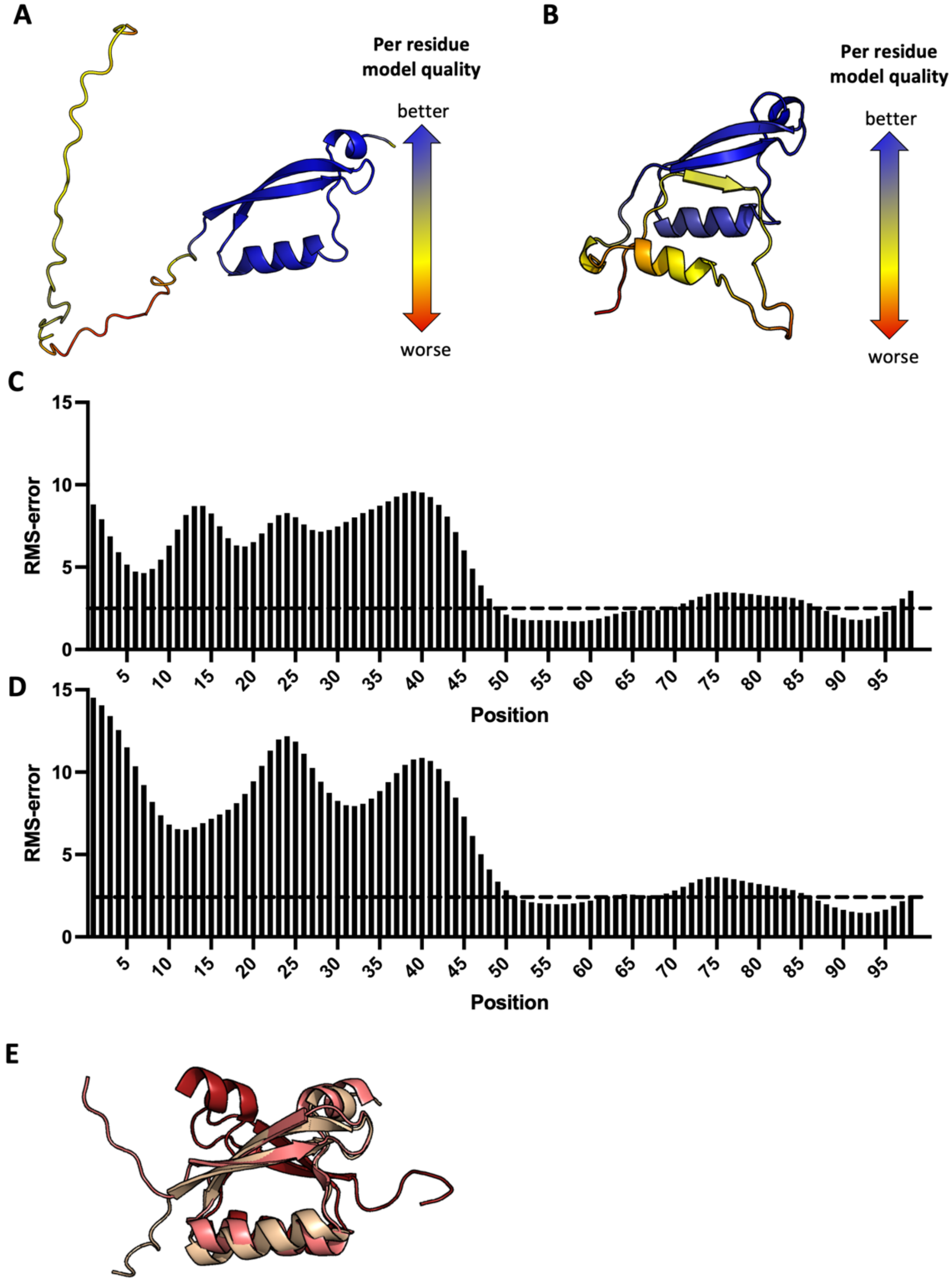

Supplementary Figure 1. Structural prediction and confidence metrics for E7 and associated designs. Additional structural prediction outputs for E7_FL, colored by confidence metrics. Red residues are lower confidence while blue residues are higher confidence. **(A)** The top ranked model from AlphaFold2, colored by per-residue pLDDT score. All AlphaFold2 models predicted an unstructured N-terminal domain; **(B)** The fourth ranked model from RoseTTaFold2, colored

by per-residue RMS-error. This model was included because it suggested an alternative conformation for the N-terminal domain; Examples of confidence metric graphs. Per residue RMS-error for E7_FL from (**C)** the top ranked RoseTTaFold2 model; **(D)** the fourth ranked RoseTTaFold2 model; **(E)** Comparison of E7_Tr to partial structure 2F8B. The partial NMR structure of HPV45 E7 (individual subunits shown in dark red and pink) is aligned to the designed foldable domain HPV16 E7_Tr (tan). Cartoon representation was used. E7_Tr has very similar folds to a single subunit of the HPV45 E7 dimer.

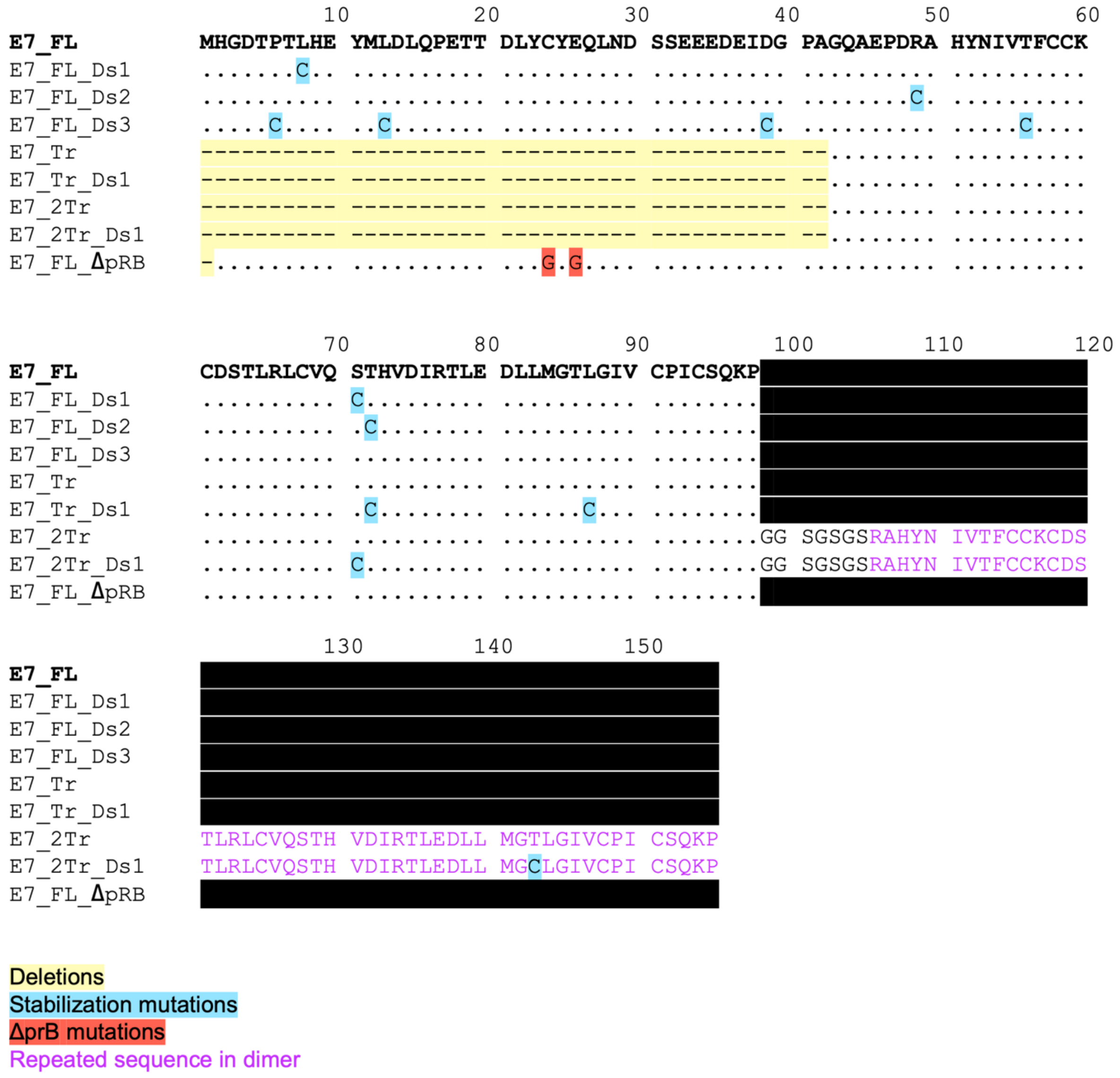

Supplementary Figure 2. Sequence alignment for E7 designs**.** All designs were aligned relative to the WT HPV16 E7 sequence (E7_FL). Dots represent identical amino acids.

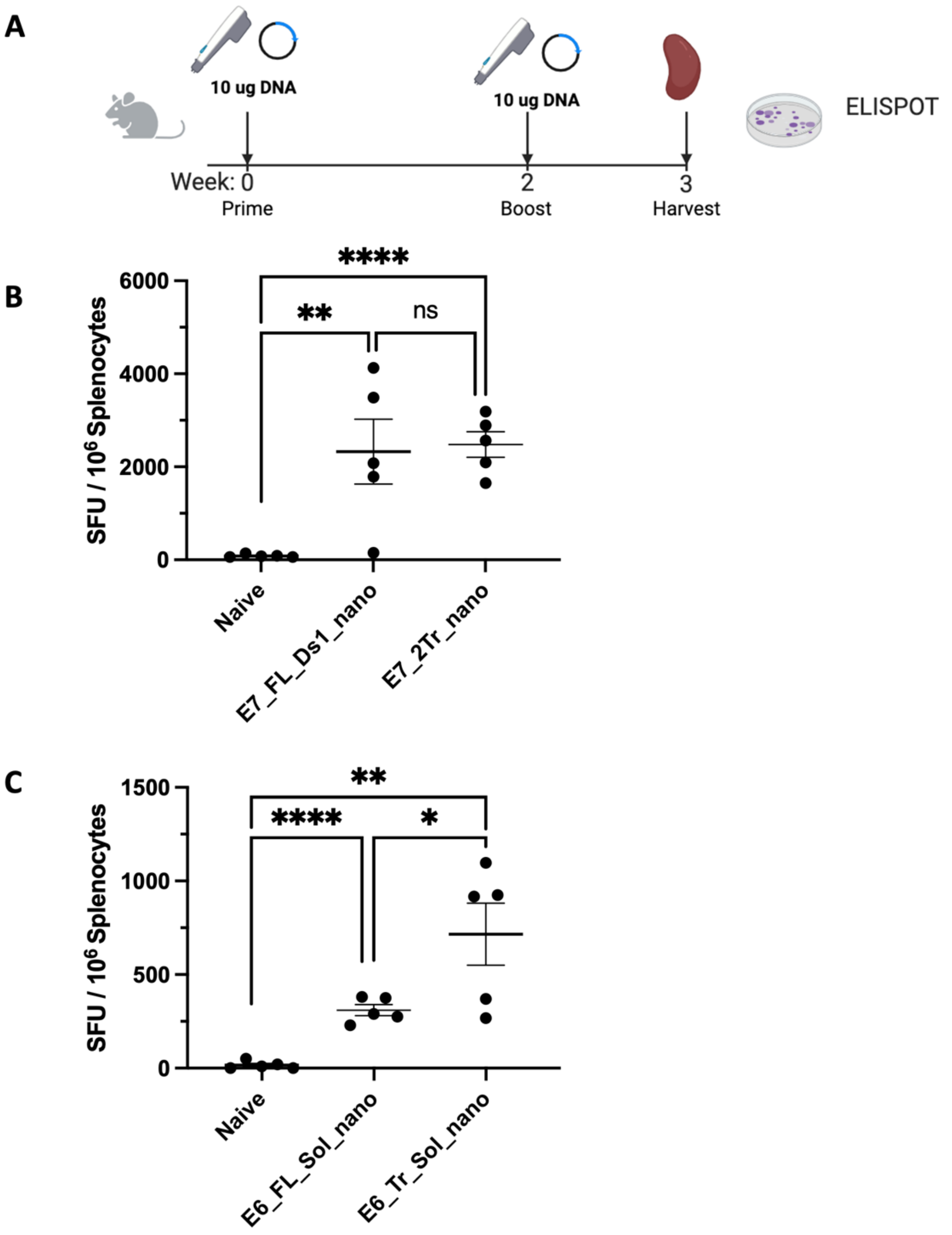

Supplementary Figure 3. Immunogenicity of first-pass E7 and E6 nanoparticle designs. **(A)** Immunizations overview. C57/BL6 were immunized with 10μg of DNA (IM-EP) at week 0. Animals were boosted with an equivalent dose at week 2, and their spleens were harvested at week 3. Splenocytes were isolated for downstream ELISpot analysis; **(B)** ELISpot of splenocytes stimulated with pooled overlapping peptides of WT HPV16 E7. n=5 mice/group. Mean ± SEM shown. One-sided T-tests were conducted. **p<0.005, ****p<0.00005; **(C)** ELISpot of splenocytes stimulated with pooled overlapping peptides (peptides 1-11) of WT HPV16 E6. n=5 mice/group. Mean ± SEM shown. One-sided T-tests were conducted. *p<0.05, **p<0.005, ****p<0.00005

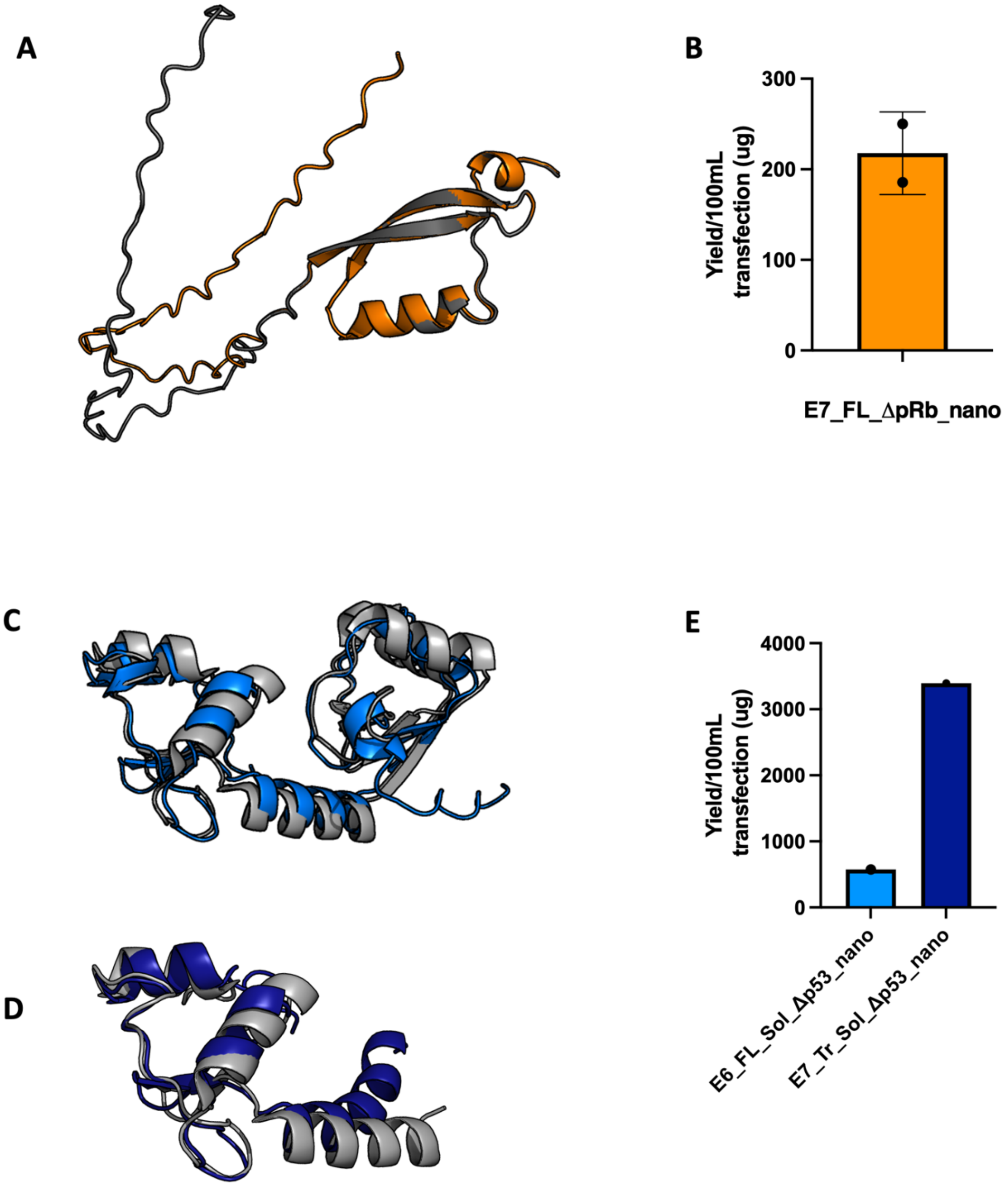

Supplementary Figure 4. Characterization of designs for safer E7 and E6 nanoparticles. **(A)** Alignment of top ranked Alphafold2 model of E7_FL_ΔpRb (orange) to top ranked Alphafold2 model of E7_FL (gray). Cartoon representation was used. The N terminal domain is predicted to be unstructured in either case, and introduction of the ΔpRb mutations does not perturb folding of the well-structured C-terminal domain; **(B)** Purified transfection yield for E7_FL_ΔpRb_nano. Transfection volume was 100mL or 300mL and constructs were purified by lectin affinity chromatography and size exclusion chromatography before yield was determined; **(C)** Alignment of top ranked Alphafold2 model of E6_FL_Sol_Δp53 (light blue) to E6 from PDB: 6SJA (gray). Cartoon representation was used. E6_FL_Sol_Δp53 shows similar shape to the crystal structure; **(D)** Alignment of top ranked Alphafold2 model of E6_Tr_Sol_Δp53 (dark blue) to E6 from

PDB: 6SJA (gray), truncated to similar residues. Cartoon representation was used. E6_Tr_Sol_Δp53 shows similar shape to the truncated crystal structure, with main differences in the position of the C-terminal alpha helix; **(E)** Purified transfection yield for E6_FL_Sol_Δp53_nano and E6_Tr_Sol_Δp53_nano. Transfection volume was 100mL and constructs were purified by lectin affinity chromatography and size exclusion chromatography before yield was determined.

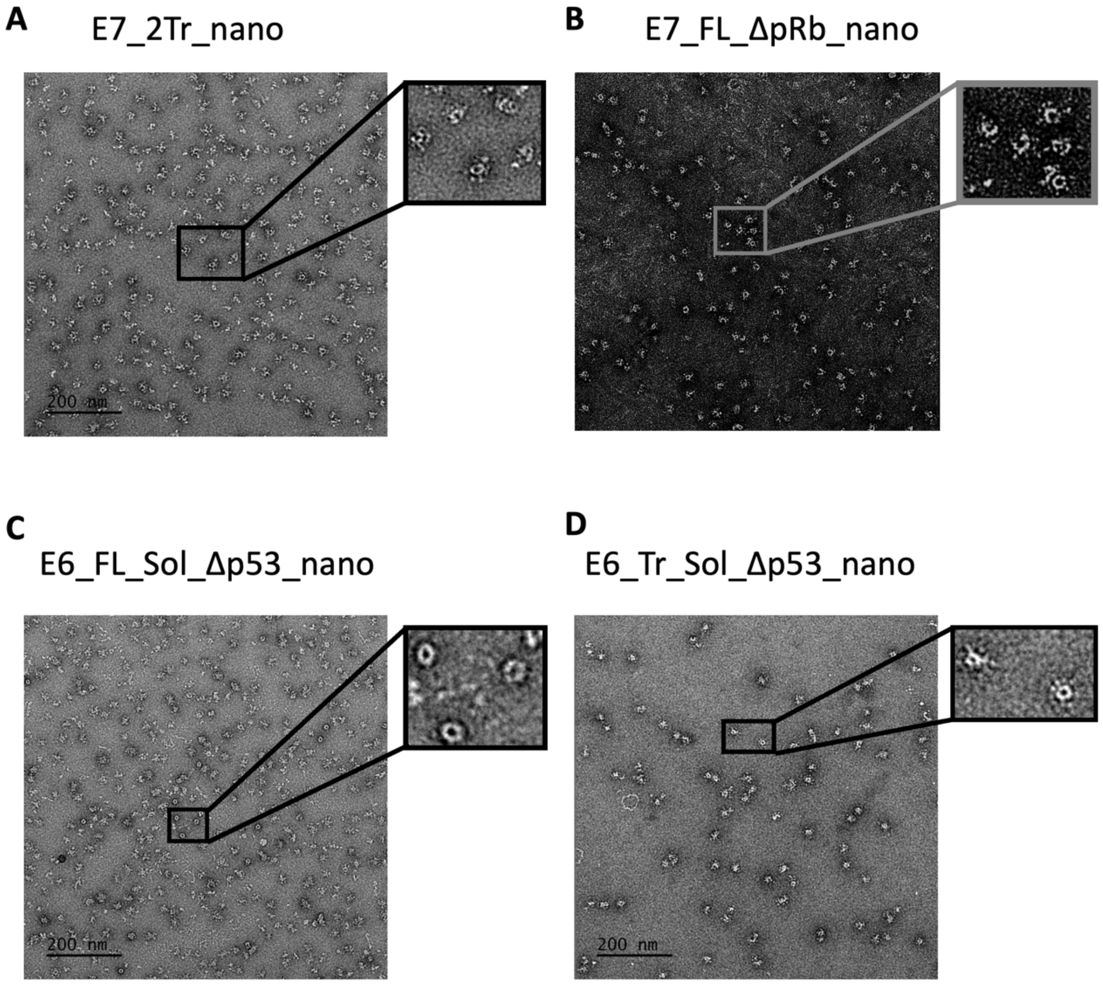

Supplementary Figure 5. Negative stain electron microscopy of lectin and SEC purified E6 and E7 lead nanoparticle designs. Left image is raw micrograph with scale bar and a corresponding zoomed in view on right to show representative nanoparticles **(A)** E7_2Tr_nano; **(B)** E7_FL_ΔpRb_nano; **(C)** E6_FL_Sol_Δp53_nano; **(D)** E6_Tr_Sol_Δp53_nano

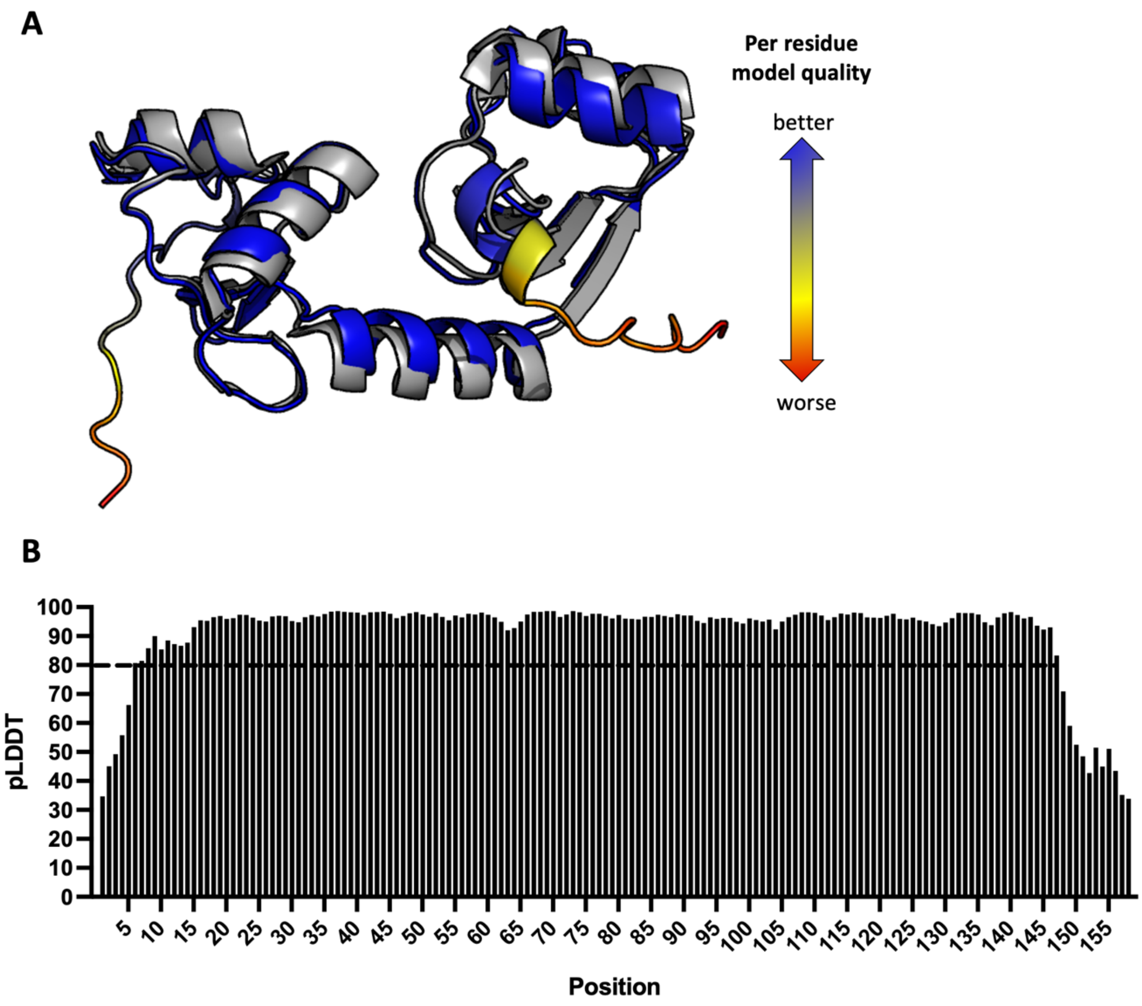

Supplementary Figure 6. Structural prediction and confidence metrics for E6_FL. **(A)** Alignment of E6 crystal structure 6SJA (gray; contains Sol muts) to top ranked Alphafold2 model of WT E6_FL (colored by confidence metrics). Red residues are lower confidence while blue residues are higher confidence. Cartoon representation was used; **(B)** Confidence metric graph. pLDDT for E6_FL from top ranked Alphafold2 model; pLDDT above 80 is considered an indication of strong model confidence.

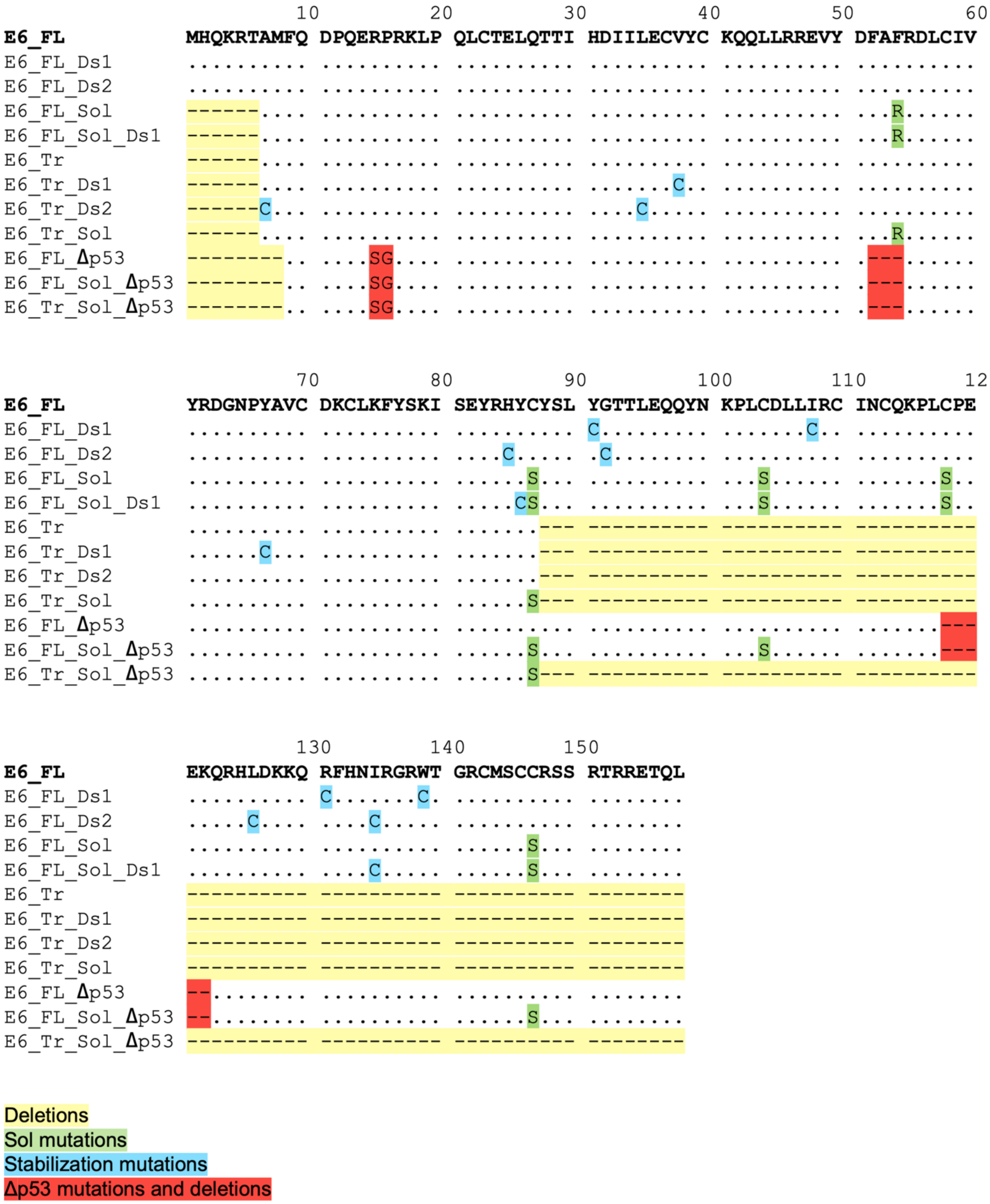

Supplementary Figure 7. Sequence alignment for E6 designs. All designs were aligned relative to the WT HPV16 E6 sequence (E6_FL). Dots represent identical amino acids.

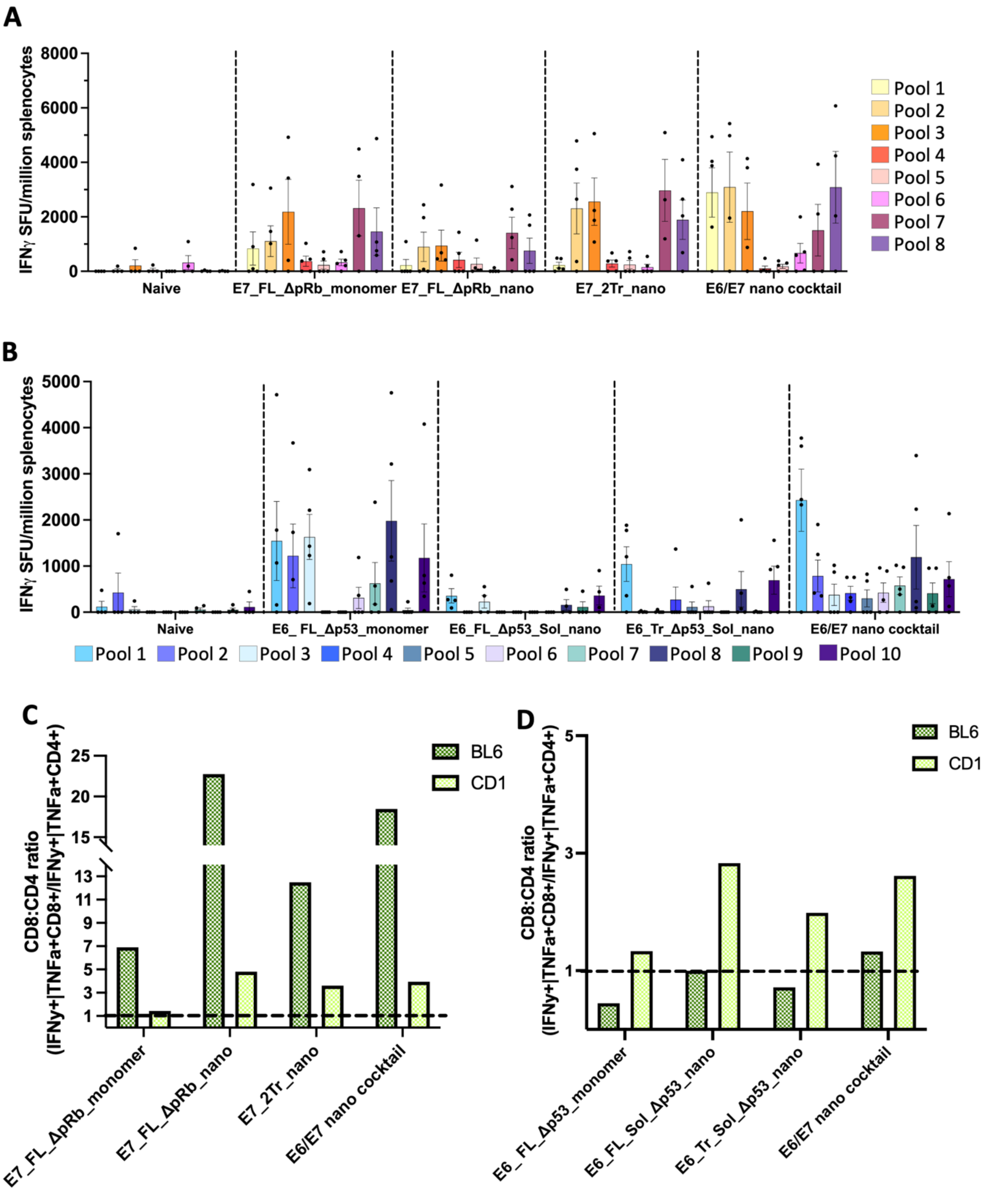

Supplementary Figure 8. ELISpot responses (outbred mice) and $CD8^+$:$CD4^+$ T-cell ratios for designed nanoparticles. **(A)** E7 ELISpot responses in outbred CD-1 mice. Splenocytes from immunized mice were stimulated with overlapping peptide pools that span WT E7. IFNγ spot forming units per million splenocytes were quantified. (n=5 mice/group). Mean ± SEM. One mouse in the naïve group responded strongly to all peptide pools and was excluded from analysis due to this nonspecific response; **(B)** E6 ELISpot responses in outbred CD-1 mice. Splenocytes

from immunized mice were stimulated with overlapping peptide pools that span WT E6. IFNγ SFU spot forming units per million splenocytes were quantified. (n=5 mice/group). Mean ± SEM; $CD8^+$:$CD4^+$ T-cell ratios. Ratios were determined as the ratio of average IFNγ$^+$ or TNFα$^+$ $CD8^+$ T-cell responses to average IFNγ$^+$ or TNFα$^+$ $CD4^+$ T-cell responses for both C57/BL6 (dark green) and CD-1 (light green) mice.**(C)** $CD8^+$:$CD4^+$ ratios for mice immunized with E7 groups; **(D)** $CD8^+$:$CD4^+$ ratios for mice immunized with E6 groups.

A

	E7 Immunodominant peptide nano	E7_2Tr_nano	E7_FL_ΔpRb_nano
HLA-A02:01	YMLDLQPET	YMLDLQPET	YMLDLQPET
	TLHEYMLDL	TLHEYMLDL	TLHEYMLDL
HLA-A01:01	QAEPDRAHY	QAEPDRAHY	QAEPDRAHY
HLA-A03:01	NA	NA	NA
HLA-A24:02	NA	NA	NA
HLA-A26:01	DTPTLHEYM	DTPTLHEYM	DTPTLHEYM
HLA-B07:02	TPTLHEYML	TPTLHEYML	TPTLHEYML
HLA-B08:01	TPTLHEYML	TPTLHEYML	TPTLHEYML
HLA-B*27:05	NA	NA	NA
HLA-B*39:01	NA	NA	NA
HLA-B40:01	LEDLLMGTL	LEDLLMGTL	LEDLLMGTL
HLA-B*58:01	RAHYNIVTF	RAHYNIVTF	RAHYNIVTF
HLA-B15:01	RAHYNIVTF	RAHYNIVTF	RAHYNIVTF
	LQPETTDLY	LQPETTDLY	LQPETTDLY
	GQAEPDRAH	GQAEPDRAH	GQAEPDRAH

B

	E6 Immunodominant peptide nano	E6_Tr_Sol_Δp53_nano	E6_FL_Sol_Δp53_nano
HLA-A02:01	NA	NA	NA
HLA-A01:01	ISEYRHYCY	ISEYRHY*C*Y	ISEYRHY*C*Y
HLA-A03:01	TTLEQQYNK	TTLEQQYNK	TTLEQQYNK
HLA-A24:02	CYSLYGTTL	CYSLYGTTL	*C*YSLYGTTL
HLA-A26:01	NA	NA	NA
HLA-B07:02	NA	NA	NA
HLA-B08:01	DKKQRFHNI	DKKQRFHNI	DKKQRFHNI
	ESGRKLPQL	ESGRKLPQL	ESGRKLPQL
HLA-B*27:05	QRFHNIRGR	QRFHNIRGR	QRFHNIRGR
	SRTRRETQL	SRTRRETQL	SRTRRETQL
HLA-B*39:01	YRDGNPYAV	YRDGNPYAV	YRDGNPYAV
HLA-B40:01	LEQQYNKPL	LEQQYNKPL	LEQQYNKPL
HLA-B*58:01	NA	NA	NA
HLA-B15:01	QQLLRREVY	QQLLRREVY	QQLLRREVY
	IVYRDGNPY	IVYRDGNPY	IVYRDGNPY
	KFYSKISEY	KFYSKISEY	KFYSKISEY

Supplementary Figure 9. Predicted immunogenic epitopes in humans for designed nanoparticles. Peptides predicted to bind human HLA alleles were determined using NetMHCpan using the 'HLA supertype representative' set of loci. The epitope corresponding to a strong HLA binder is specified or shown as NA if no strong binders were predicted. If that sequence is present in the designed nanoparticle, it is highlighted green to show predicted immunogenicity. Binding peptides (9-mers) were predicted from the **(A)** E7_FL_ΔpRb sequence; or **(B)** E6_FL_Δp53 sequence. Italics indicate residues that are mutated in the designed nanoparticle.
